# Mitochondrial Markers COI and 16S rRNA for the Molecular Identification of Parrots and Macaws Recovered From Illegal Trafficking in Three Areas of Colombia

**DOI:** 10.1002/ece3.73335

**Published:** 2026-03-25

**Authors:** Julián Marín‐Villa, Juan Carlos Rincón‐Flórez, Cristina Úsuga‐Monroy, Albeiro López‐Herrera

**Affiliations:** ^1^ Grupo BIOGEM, Faculty of Sciences Universidad Nacional de Colombia Medellín Colombia; ^2^ Grupo de Investigación en Recursos Zoogenéticos, Faculty of Agricultural Sciences Universidad Nacional de Colombia Palmira Colombia; ^3^ Grupo GINVER, Faculty of Veterinary Medicine Corporación Universitaria Remington Medellín Colombia; ^4^ Grupo BIOGEM, Faculty of Agricultural Sciences Universidad Nacional de Colombia Medellín Colombia

**Keywords:** DNA barcode, mtDNA, phylogeny, wildlife trafficking

## Abstract

Illegal wildlife trafficking is a major threat to biodiversity, severely affecting many species, including Psittacidae. In this context, molecular tools such as DNA barcoding provide an effective alternative for taxonomic identification, complementing traditional morphological methods. This study aimed to molecularly characterize parrots (*Amazona* spp.) and macaws (*Ara* spp.) recovered from illegal trafficking in Colombia using two mitochondrial markers (COI and 16S rRNA). Eighty‐eight DNA samples from whole blood of Psittacidae individuals from three regions (Antioquia, Valle del Cauca, and Cesar) were analyzed. The COI gene provided higher resolution for species identification, generating 65 new sequences and 35 haplotypes (20 *Amazona* spp. and 15 *Ara* spp.). Unique haplotypes were found for each species, with clear differentiation in phylogenetic and PCoA analyses. High intraspecific diversity was detected in *Amazona amazonica* , *Ara severus* , and *Ara ararauna* , relevant for future population studies. In contrast, 16S rRNA yielded 77 new sequences clustered into 28 haplotypes (19 *Amazona* spp. and 9 *Ara* spp.). However, its low variability limited taxonomic resolution, with poorly defined clusters in haplotype networks and PCoA. Phylogenetic inference supported species‐level grouping under mitochondrial data, except within the yellow‐headed parrot complex. Overall, the COI gene demonstrated greater utility for species identification and genetic characterization in *Amazona* and *Ara* species, while the 16S rRNA gene showed limited discriminatory power due to its conserved nature. These findings highlight the value of COI as a reliable molecular tool for wildlife forensic applications and strengthen molecular identification frameworks to combat illegal wildlife trafficking.

## Graphical abstract

We molecularly characterized parrots (*Amazona* spp.) and macaws (*Ara* spp.) rescued from illegal trafficking in Colombia using two mitochondrial markers (COI and 16S rRNA). The COI gene showed high resolution for species identification and revealed considerable intraspecific diversity, while 16S rRNA displayed limited taxonomic resolution. These results highlight the value of DNA barcoding for monitoring and conserving psittacine species affected by wildlife trafficking.

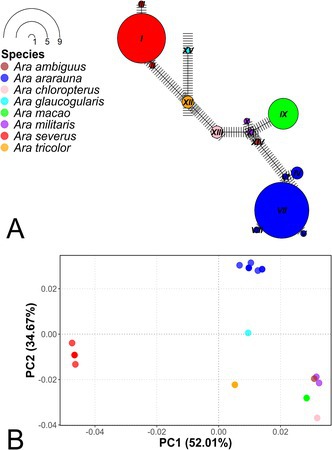

## 1 Introduction

The illegal wildlife trade is one of the main threats to biodiversity and is one of the most profitable illicit businesses worldwide, generating an estimated annual profit between $7 billion and $23 billion USD (Morton et al. 2021; Tittensor et al. 2020). This illegal activity represents a significant risk, particularly for endemic, threatened, or endangered species, making them increasingly more attractive to the black market (Burnham‐Curtis et al. 2021). In addition to direct population losses, illegal wildlife trade can lead to the loss of genetic diversity and, in the worst case, local extinctions (Mozer and Prost 2023).

This problem especially affects the most biodiverse countries, such as Colombia, which is positioned as one of the territories with the greatest biological richness in the world (Rangel 2015). This mega‐biodiversity, along with social factors such as inequality and poverty, has paved the way for the growth of this illegal activity as an alternative source of livelihood for families, generating serious consequences for wildlife populations (Carmona and Arango 2011). One of the groups most affected by this business is Psittacidae birds, such as parrots (*Amazona* spp.) and macaws (*Ara* spp.), which are highly sought after by the illegal market due to their attractiveness as pets, their striking colors, and social behaviors (Bello 2010).

In this context, molecular biology tools such as the use of DNA barcoding methodologies have emerged as an alternative to help identify and study Psittacidae birds that are highly affected by illegal trafficking, including complete specimens or byproducts derived from these (Formentão et al. 2021; Gonçalves et al. 2015). These techniques are used to achieve rapid and accurate taxonomic identification by sequencing short fragments of mitochondrial genes such as cytochrome oxidase 1 (COI) and 2 (COII), cytochrome b (Cyt b), and 16S rRNA. These genes have been used as DNA barcodes that are standard genetic markers to identify different groups of animals (Almerón et al. 2018; Carvalho 2013; Chen et al. 2023; Francis et al. 2010; Hebert et al. 2003, 2010).

Currently, in Colombia, species identification by environmental authorities is carried out mainly through morphological techniques, which have limitations in cases where the specimens are incomplete, have been transformed into derived products, or belong to a group of species that are morphologically similar (i.e., cryptic species) (Mendoza et al. 2016; Restrepo‐Rodas and Pulgarín‐Restrepo 2021). Therefore, molecular identification using DNA barcodes is positioned as a complement to traditional techniques, overcoming these limitations and offering an additional tool for complex cases.

The genetic information deposited in databases such as the Barcode of Life Data Systems (BOLD) for species of the *Amazona* and *Ara* genera is relatively limited. Currently, for these genera, sequences for 445 parrots (*Amazona* spp.) and 88 macaws (*Ara* spp.) are included (https://boldsystems.org, accessed December 12, 2024). However, these numbers are limited compared to the high biological richness and diversity of these species in Colombia, which hosts a significant proportion of the Psittacidae species found worldwide.

The genetic information deposited in databases, such as the Barcode of Life Data Systems (BOLD) for species of the *Amazona* and *Ara* genera is relatively limited. The genus *Amazona* comprises approximately 31 recognized species worldwide, of which 6 occur in Colombia, while the genus *Ara* includes 9 species globally, with 6 present in Colombia (https://www.worldbirdnames.org/new/bow/parrots/, accessed January 07, 2025). BOLD System includes sequences from 445 individuals of *Amazona* spp. and 88 individuals of *Ara* spp. (https://boldsystems.org, accessed December 12, 2024). Additionally, GenBank (NCBI) includes 109 publicly available 16S rRNA sequences for *Amazona* and 170 for *Ara* (https://www.ncbi.nlm.nih.gov, accessed December 12, 2024). These records represent only a fraction of the recognized species diversity within each genus and show uneven taxonomic and geographic representation, particularly for Colombian populations, despite the country hosting a substantial proportion of the global diversity of these genera.

This study provides a novel regional contribution to the molecular characterization of Psittacidae species affected by illegal trafficking in Colombia. In contrast to previous barcoding efforts (Mendoza et al. 2016; Arias‐Sosa et al. 2024), our dataset includes newly generated sequences for both COI and 16S rRNA markers, representing individuals recovered from three regions (Antioquia, Valle del Cauca, and Cesar). To our knowledge, this is the first study to combine dual mitochondrial markers and multiregional sampling for confiscated *Amazona* and *Ara* species in Colombia, expanding the available genetic reference data for these genera.

Accordingly, this study aims to characterize parrots and macaws using two mitochondrial markers (COI and 16S rRNA) recovered from illegal trafficking in three regions of Colombia.

## 2 Materials and Methods

### 2.1 Ethical Considerations

This study is part of the macroproject “Genomic evaluation and positivity to *Chlamydia psittaci* for the management of parrots and macaws recovered from trafficking in three areas of Colombia,” which has the ethical endorsement of the Institutional Committee for the Care and Use of Animals (CICUA, for its Spanish acronym) of Universidad Nacional de Colombia, Medellín campus, in its Act 13 of 2023. The Wildlife Care and Assessment Centers (CAV, for its Spanish acronym) participating in the study signed the corresponding informed consents, ensuring compliance with ethical and legal regulations.

### 2.2 Study Area and Sample Collection

**FIGURE 1:**
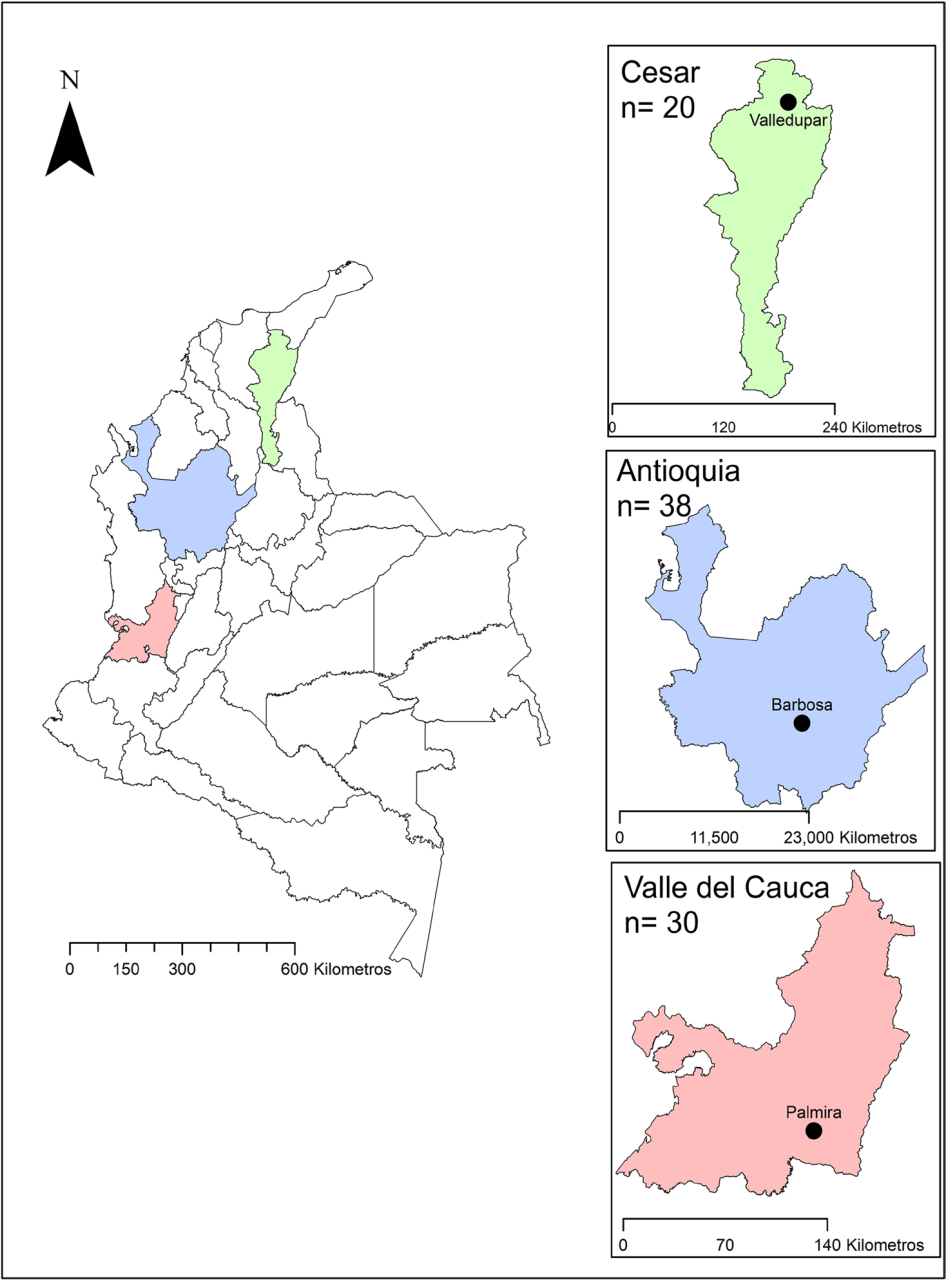
Geographic distribution of sampling regions and number of individuals sampled per region.

Sampling was carried out between December 2023 and June 2024 in the CAVs located in three regions of Colombia (Figure 1): Barbosa (Antioquia), Valledupar (Cesar), and Palmira (Valle del Cauca). This study was descriptive and observational, using non‐probability convenience sampling, including all Psittacidae individuals that entered the CAVs through confiscations because of illegal trafficking during the study period. A total of 88 recovered individuals were included, of which 57 belonged to the *Amazona* genus and 31 to the genus *Ara*.

Additionally, a morphological taxonomic identification was carried out by the veterinary medical staff and biologists of each CAV as part of the routine procedure for the admission of individuals. This initial classification served as the basis for the molecular validation of the species included in the study.

Figure 1 shows a distribution map depicting the three sampling regions (Antioquia, Valle del Cauca, and Cesar) and the total number of individuals collected per area. A total of 88 individuals were included, of which 57 belong to the genus *Amazona* and 31 to the genus *Ara*.

For each individual, 0.5 mL of blood was collected by wing venipuncture. The samples were stored in microtubes with EDTA as an anticoagulant and homogenized by manual inversion. They were then transported under refrigeration conditions in polystyrene coolers with cooling gel. The samples collected in the department of Antioquia were sent for processing to the Animal Biotechnology Laboratory of Universidad Nacional de Colombia, Medellín campus; those obtained in Cesar were processed in the Molecular Biology Laboratory of Universidad Nacional de Colombia, La Paz campus; and those from Valle del Cauca were processed in the Molecular Biology Laboratory of Universidad Nacional de Colombia, Palmira campus.

### 2.3 DNA Extraction, Amplification and Sanger Sequencing

Genomic DNA extraction in all cases was performed using the HigherPurity Blood and Cell Culture DNA Extraction kit (CANVAX Biotech, Spain), following the manufacturer's specifications. However, the initial protocol was adjusted by reducing the sample volume of whole blood to 25 μL and completing it with PBS to a total volume of 250 μL. DNA elution was carried out in a final volume of 30 μL of elution buffer. The quality and quantity of extracted DNA were analyzed in a Nanodrop 2000 spectrophotometer (Thermo Fisher Scientific).

#### 2.3.1 Partial Amplification of the COI Mitochondrial Gene

A partial fragment of the COI mitochondrial gene with a band size of 960 bp was amplified using two primers, LTyr (5′‐TGTAAAAAGGWCTACAGCCTAACGC‐3′) and COIH7557 (5′‐GGCGGATGTGGAAGTATGCTCGGG‐3′) (Tavares and Baker 2008). All PCR reactions were performed at the Animal Biotechnology Laboratory of Universidad Nacional de Colombia, Medellín campus, using a MultigeneTM Optimax thermal cycler. The PCR reaction was carried out in a final volume of 50 μL, which contained 1 μL of DNA (75 ng/μL), 0.5 μL of each primer (10 mM), 0.5 μL of dDNTPs (25 mM), 5 μL of 10× Taq Buffer (ExcelTaq Taq Polymerase, Smobio), 0.25 μL of Taq Polymerase (5 U/μL), and 42.25 μL of water. A negative control with molecular‐grade water and a positive control corresponding to the DNA of an individual of the species *Ara macao* from Parque de la Conservación in Medellin, Colombia, previously amplified and verified, were included. The thermal profile used was as follows: initial denaturation at 95°C for 5 min, 35 cycles of denaturation at 95°C for 30 s, annealing at 63°C for 30 s, extension at 72°C for 40 s, and a final extension at 72°C for 10 min. The PCR product was visualized by 1.2% agarose gel electrophoresis using a Gel Doc XR System photodocumenter (BioRad); this equipment was used in all visualizations.

#### 2.3.2 Partial Amplification of 16S rRNA Mitochondrial Gene

A partial fragment of approximately 540 bp of the 16S rRNA mitochondrial gene was amplified using two primers, 16Sar‐L (5′‐CGCCTGTTTATCAAAAACAT‐3′) and 16Sbr‐H (5′‐CCGGTCTGAACTCAGATCACGT‐3′) (Palumbi et al. 2002). All PCR reactions were performed at the Animal Biotechnology Laboratory of Universidad Nacional de Colombia, Medellín campus, using a thermocycler Multigene Optimax. The PCR reaction was performed in a final volume of 30 μL, containing 1 μL of DNA (75 ng/μL), 0.5 μL of each primer (10 mM), 0.5 μL of dDNTPs (25 mM), 3 μL of 10× Taq Buffer (ExcelTaq Taq Polymerase, Smobio), 0.25 μL of Taq Polymerase (5 U/μL), and 22.25 μL of water. A negative control with molecular‐grade water and a positive control corresponding to the DNA of an individual of the species *Ara macao* from Parque de la Conservación in Medellin, Colombia, previously amplified and verified, were included. The thermal profile consisted of an initial denaturation of 94°C for 2 min, followed by 35 cycles of denaturation at 94°C for 30 s, annealing at 53°C for 30 s, extension at 72°C for 48 s, and a final extension at 72°C for 1 min. PCR products were visualized as described in section 2.5.1.

#### 2.3.3 Sanger Sequencing

The amplified PCR products were sent to Macrogen (Korea) for purification and subsequent Sanger sequencing in both directions (forward and reverse). Samples of 20 μL of the amplified PCR products were sent for this procedure. Chromatograms were visualized and edited manually using the FinchTV v.1.4.0 software (Geospiza Inc., Seattle, WA, USA), and consensus sequences were generated using the Cap3 tool (Huang and Madan 1999).

### 2.4 Data Analysis

#### 2.4.1 Characterization of Populations

The sampled populations were characterized in each of the three study regions (i.e., Antioquia, Cesar, and Valle del Cauca) based on the distribution of species identified in each area. The JASP software (Love et al. 2019) was used for the descriptive analysis.

#### 2.4.2 Genetic Distances

For the genetic analyses, additional reference sequences were retrieved from public databases. Sequences of the 16S rRNA gene were obtained from the NCBI database, while COI sequences were retrieved from both the NCBI and BOLD Systems databases. Sequences from other species belonging to the genus *Amazona* and *Ara* were incorporated to achieve the broadest possible taxonomic coverage. These reference sequences were included together with those generated in this study in all subsequent analyses, including genetic distance estimates, phylogenetic inference, and genetic diversity analyses. External sequences were selected from GenBank and BOLD to match the species analyzed in this study and to include additional representatives of both genera, ensuring broad phylogenetic representation. Sequence quality was verified by inspecting chromatograms and confirming Phred scores above 30 before inclusion; terminal regions with ambiguous bases or low‐quality sites were manually trimmed before downstream analyses in MEGA v11 (Tamura et al. 2021). Reference COI and 16S rRNA sequences retrieved from GenBank and BOLD Systems used in the analyses are listed in Tables S1 and S2.

Prior to downstream analyses, all COI and 16S rRNA sequences were aligned using the MUSCLE algorithm implemented in MEGA v11 (Tamura et al. 2021). The same datasets were used for all analyses to ensure methodological consistency. To ensure comparability between markers, COI alignments were standardized to 610 bp and 16S rRNA alignments to 475 bp.

Mean interspecific genetic distances were estimated using the Kimura 2‐parameter (K2P) model in MEGA v11 (Tamura et al. 2021), with species defined as groups to assess genetic divergence among species within each genus.

#### 2.4.3 Phylogenetic Inference

The IQ‐TREE v2.3.1 software (Minh et al. 2020) was used to reconstruct phylogenetic trees using the Maximum Likelihood (ML) method with 1000 bootstrap replicates. The best‐fit evolutionary model for each dataset was independently selected using ModelFinder as implemented in IQ‐TREE under the Akaike Information Criterion (AIC). The selected models were HKY + F + G4 for COI in *Amazona*, HKY + F + I for 16S in *Amazona*, TN + F + G4 for COI in *Ara*, and HKY + F + I for 16S in *Ara*. The resulting trees were visualized and edited with the iTOL v6 software (Letunic and Bork 2024).

#### 2.4.4 Genetic Diversity

Genetic diversity analyses were performed using the R software (http://www.r‐project.org/). Genetic diversity was assessed using the *F*_st_ statistic, expected heterozygosity of the subpopulation (*H*_s_), and expected heterozygosity of the total population (*H*_t_), using the *hierfstat* package (Goudet 2005). The *F*_st_ values were estimated with the Reynolds method, while *H*_s_ and *H*_t_ were calculated using the *basis.stats* function.

For the haplotype network analysis, previously aligned sequences were processed with the haplotype function of the *pegas* package (Paradis 2010) to identify the different haplotypes. The Hamming genetic distance between haplotypes was calculated using the *dist.dna* function of the *ape* package (Paradis and Schliep 2019). With the distance matrix obtained, haplotype networks were constructed using the *haploNet* function, which allowed visualizing the relationships and genetic variability between the haplotypes of the analyzed species. Additionally, haplotype frequencies were evaluated by species using the *haploFreq* function. The *ape* package was used to carry out a principal coordinates analysis (PCoA) (Paradis and Schliep 2019) to calculate the distance matrix based on the F81 substitution model. Subsequently, a principal coordinate analysis was performed using the *pcoa* function. The results obtained were graphically represented using the *ggplot2* library, allowing the visualization of the species distribution in the space defined by the first two principal components. This analysis was carried out to identify patterns of genetic variability and possible structuring in the evaluated species.

## 3 Results

### 3.1 Distribution of the Birds Evaluated by Region and Species

Table 1 presents the general distribution results of the evaluated birds. Within the genus *Amazona* (*n* = 57), the species *Amazona ochrocephala* was the most frequent, with 35 samples (39.8%) distributed between Antioquia (14.8%), Valle del Cauca (14.8%), and Cesar (10.2%). The species *Amazona amazonica* followed, representing 17% of the samples, being observed in greater proportion in Antioquia (13.6%) and Valle del Cauca (3.4%). This species was not present in Cesar. Other species, such as *Amazona autumnalis* (6.8%) and one individual identified at the genus level as *Amazona* sp. (1.1%), were also present; the morphological identification of this individual to the species level was not possible, and therefore, it was identified only to the genus level.

**TABLE 1:** Frequency (in number and percentage) and distribution of species of the *Amazona* and *Ara* genera by region.

Genus	Species	CAVR Barbosa (Antioquia) (*n*)	CAV Palmira (Valle del Cauca) (*n*)	CAVFF Valledupar (Cesar) (*n*)	Total (*n*)	Total (%)
*Amazona*	*amazonica*	12 (13.6%)	3 (3.4%)	0 (0.0%)	15	17.0
	*autumnalis*	3 (3.4%)	3 (3.4%)	0 (0.0%)	6	6.8
	*ochrocephala*	13 (14.8%)	13 (14.8%)	9 (10.2%)	35	39.8
	*Amazona* sp.	0 (0.0%)	1 (1.8%)	0 (0.0%)	1	1.1
Frequency by genus		28 (31.8%)	20 (22.7%)	9 (10.7%)	57	64.8
*Ara*	*ararauna*	4 (4.5%)	4 (4.5%)	11 (12.5%)	19	21.6
	*macao*	1 (1.1%)	3 (3.4%)	0 (0.0%)	4	4.5
	*militaris*	1 (1.1%)	0 (0.0%)	0 (0.0%)	1	1.1
	*severus*	4 (4.5%)	3 (3.4%)	0 (0.0%)	7	8.0
Frequency by genus		10 (11.4%)	10 (11.4%)	11 (12.5%)	31	35.2
General frequency		38 (43.2%)	30 (34.1%)	20 (22.7%)	88	100

The *Ara* genus (*n* = 31) contributed 35.2% of the total samples, with *Ara ararauna* being the most common species (21.6%), concentrated mainly in Cesar (12.5%), followed by Antioquia (4.5%) and Valle del Cauca (4.5%). Less representative species included *Ara severus* (8.0%), *Ara macao* (4.5%), and *Ara militaris* (1.1%), with varying presence across the regions studied.

At the regional level, Antioquia registered the largest number of samples (43.2%), followed by Valle del Cauca (34.1%) and Cesar (22.7%). Species of the *Amazona* genus predominated in Antioquia and Valle del Cauca, while a greater representation of the genus *Ara* was observed in Cesar.

#### 3.1.1 Sanger Amplification and Sequencing

Of the 88 samples processed, successful amplification and sequencing of the COI and 16S rRNA genes were achieved in most individuals. For the COI gene fragment, with a band size of 960 bp (Figure S1), partial sequences were obtained in 65 individuals, of which 40 corresponded to the genus *Amazona* and 25 to the genus *Ara*.

On the other hand, for the amplified fragment of the 16S rRNA gene, with a band size of 540 bp (Figure S2), 77 individuals were successfully amplified and sequenced, with 49 sequences belonging to the genus *Amazona* and 28 to the genus *Ara*. Amplification success was not completely overlapping between loci, as some individuals yielded sequences for only one of the two markers.

#### 3.1.2 Basic Statistics and Genetic Diversity Indices

As shown in Table 2, the analysis of the alignments showed that the COI gene presented greater variability compared to 16S rRNA, evidenced by a higher number of polymorphic and parsimoniously informative sites in both genera. In contrast, 16S rRNA presented a higher number of conserved sites, making it a gene with less variability.

**TABLE 2:** Basic statistics from the COI and 16S rRNA gene sequences in individuals of the *Amazona* and *Ara* genera.

	*Amazona* spp.		*Ara* spp.	
	COI gene	16S rRNA gene	COI gene	16S rRNA gene
Alignment length (bp)	610	475	610	475
Conserved sites	504	428	501	431
Variable sites	106	47	109	44
Parsimoniously informative sites	76	28	81	26
Singleton	30	19	28	18
Nucleotide composition (%)				
Adenine (A)	27.2	33.3	25.1	31.2
Thymine (T)	22.0	17.4	25.1	17.9
Cytosine (C)	34.9	29.4	32.1	29.6
Guanine (G)	15.9	19.9	17.7	21.3

Genetic differentiation and diversity indices are provided in Table S3. *F*_st_ values were considerably higher for the COI gene in both genera (*Amazona* = 0.9608, *Ara* = 0.9630), indicating a strong genetic differentiation between the analyzed species. In the case of the 16S rRNA gene, the *Amazona* genus presented a low *F*_st_ (0.0017), which can be attributed to the conserved nature of this gene, while *Ara* showed an *F*_st_ of 0.7455, indicating genetic differentiation within this genus.

The expected heterozygosity (*H*_s_) of the COI gene for the genus *Amazona* was 0.0128 and 0.0113 for the *Ara* genus. However, for the 16S rRNA gene, the *Amazona* genus had a higher *H*_s_ (0.1663) compared to *Ara* (0.0767).

#### 3.1.3 Genetic Distances and Reynolds' Paired *F*_st_

##### 3.1.3.1 Genetic Distances of the Amazona Genus for the COI and 16S rRNA Genes

The highest divergence for the *Amazona* genus for the COI gene was found between *Amazona ventralis* and *Amazona ochrocephala* (0.076), indicating a high level of genetic differentiation between these species. The smallest distance was observed between *Amazona barbadensis* and *Amazona aestiva* (0.009), suggesting a closer genetic relationship between them (Table 3).

**TABLE 3:** Estimates of genetic distances (evolutionary divergence) between pairs of sequences of the *Amazona* genus for the COI gene, according to the Kimura 2‐Parameter (K2P) model and Reynolds' pairwise *F*_st_.

Distance between species and Reynolds' paired *F*_st_									
COI gene of the *Amazona* genus									
*Amazona* spp.	1	2	3	4	5	6	7	8	9
1. *A. ventralis*		0.971	0.992	1.000	0.972	0.996	0.994	1.000	0.991
2. *A. leucocephala*	0.014		0.985	0.993	0.965	0.990	0.988	0.994	0.985
3. *A. finschi*	0.055	0.060		0.992	0.961	0.984	0.983	0.992	0.980
4. *A. guildingii*	0.064	0.063	0.057		0.928	0.996	0.992	1.000	0.990
5. *A. amazonica*	0.069	0.068	0.060	0.025		0.967	0.965	0.970	0.961
6. *A. autumnalis*	0.063	0.068	0.040	0.049	0.065		0.988	0.996	0.985
7. *A. aestiva*	0.074	0.075	0.054	0.059	0.068	0.057		0.955	0.925
8. *A. barbadensis*	0.073	0.072	0.053	0.062	0.063	0.060	0.009		0.945
9. *A. ochrocephala*	0.076	0.075	0.054	0.065	0.066	0.059	0.014	0.011	

*Note:* The lower diagonal contains the genetic distances between species, while the upper diagonal presents Reynolds' pairwise *F*_st_ values.

Regarding *Amazona ochrocephala* , *Amazona aestiva* , and *Amazona barbadensis* , genetic distances were low, with values of 0.009 between *Amazona barbadensis* and *Amazona aestiva* , 0.011 between *Amazona barbadensis* and *Amazona ochrocephala* , and 0.014 between *Amazona aestiva* and *Amazona ochrocephala* ; these results suggest that this group of species could be closely related.

The genetic distances found for the 16S rRNA gene in the *Amazona* genus are lower than those obtained for the COI gene. The species with the greatest divergence were *Amazona ventralis* and *Amazona vinacea* (0.038), *Amazona vinacea* and *Amazona leucocephala* (0.038), *Amazona amazonica* and *Amazona ventralis* (0.038), and *Amazona amazonica* and *Amazona leucocephala* (0.038). In contrast, the species with the lowest genetic distances were *Amazona auropalliata* and *Amazona barbadensis* (0.002), *Amazona ochrocephala* and *Amazona auropalliata* (0.004), *Amazona leucocephala* and *Amazona ventralis* (0.004), and *Amazona amazonica* and *Amazona guildingii* (0.004) (see Table 4). Overall, the genus *Amazona* shows lower genetic distances in the 16S rRNA gene compared to the COI gene. These results are consistent with the values obtained in Table 2, where the 16S rRNA gene showed a lower number of polymorphic and parsimoniously informative sites, as well as a higher proportion of conserved sites compared to COI.

**TABLE 4:** Estimates of genetic distances (evolutionary divergence) between pairs of sequences of the *Amazona* genus for the 16S rRNA gene, according to the Kimura 2‐Parameters (K2P) model and Reynolds' paired *F*_st_.

Distance between species and Reynolds' paired *F*_st_											
16S rRNA gene of the *Amazona* genus											
*Amazona* spp.	1	2	3	4	5	6	7	8	9	10	11
1. *A. ventralis*		0.433	0.367	0.833	0.833	0.558	0.982	0.833	0.833	0.900	0.305
2. *A. leucocephala*	0.004		0.353	0.833	0.667	0.557	0.983	0.667	0.667	0.867	0.257
3. *A. autumnalis*	0.031	0.031		0.443	0.398	0.188	0.640	0.398	0.398	0.443	0.210
4. *A. vinacea*	0.038	0.038	0.028		1.000	0.612	1.000	1.000	1.000	1.000	0.440
5. *A. guildingii*	0.034	0.034	0.028	0.026		0.593	1.000	0*	0*	1.000	0.284
6. *A. amazonica*	0.038	0.038	0.027	0.030	0.004		0.678	0.593	0.593	0.593	0.342
7. *Amazona* sp.	0.031	0.031	0.028	0.033	0.029	0.028		1.000	1.000	1.000	0.797
8. *A. aestiva*	0.027	0.027	0.021	0.027	0.027	0.031	0.034		0*	1.000	0.284
*9. A. barbadensis*	0.029	0.029	0.021	0.029	0.024	0.028	0.031	0.015		1.000	0.284
10. *A. auropalliata*	0.027	0.027	0.019	0.027	0.022	0.026	0.029	0.013	0.002		0.440
11. *A. ochrocephala*	0.023	0.023	0.020	0.024	0.021	0.025	0.028	0.010	0.006	0.004	

*Note:* 0* = value that could not be calculated because there was only one individual of the species and was represented with the same haplotype. The lower diagonal contains the genetic distances between species, while the upper diagonal presents Reynolds' paired *F*_st_ values.

##### 3.1.3.2 Genetic Distances of the Ara Genus for the COI and 16S rRNA Genes

The highest genetic divergence for the COI gene in the *Ara* genus was found between *Ara severus* and *Ara chloropterus* (0.095), indicating considerable genetic differences between these two species and suggesting that they are quite distant evolutionarily. Likewise, the lowest genetic divergence was observed between *Ara ambiguus* and *Ara militaris* (0.010) (see Table 5).

**TABLE 5:** Estimates of genetic distances (evolutionary divergence) between pairs of sequences of the *Ara* genus for the COI gene, according to the Kimura 2‐Parameters (K2P) model and Reynolds' paired *F*_st_.

Distance between species and Reynolds' paired *F*_st_								
COI gene for the *Ara* genus								
*Ara* spp.	1	2	3	4	5	6	7	8
1. *A. severus*		0.969	0.988	0.967	0.985	0.989	0.988	0.988
2. *A. ararauna*	0.074		0.982	0.950	0.980	0.984	0.979	0.982
*3. A. macao*	0.085	0.067		0.944	1.000	1.000	1.000	1.000
4. *A. militaris*	0.088	0.061	0.031		0.963	0.952	0.833	0.975
*5. A. tricolor*	0.065	0.062	0.050	0.048		1.000	1.000	1.000
6. *A. chloropterus*	0.095	0.079	0.043	0.037	0.043		1.000	1.000
7. *A. ambiguus*	0.086	0.059	0.029	0.043	0.037	0.069		1.000
8. *A. glaucogularis*	0.086	0.067	0.073	0.075	0.063	0.084	0.010	

*Note:* The lower diagonal contains the genetic distances between species, while the upper diagonal presents the Reynolds' paired *F*_st_ values.

Regarding the 16S rRNA gene, the highest divergence was found between *Ara chloropterus* and *Ara severus* (0.046). On the other hand, the lowest divergence was observed between *Ara ambiguus* and *Ara militaris* (0.002) (Table 6). As in the *Amazona* genus, the genetic distances for the 16S rRNA gene in the *Ara* genus are smaller compared to those obtained for the COI gene.

**TABLE 6:** Estimates of genetic distances (evolutionary divergence) between pairs of sequences of the *Ara* genus for the 16S rRNA gene, according to the Kimura 2‐Parameters (K2P) model and Reynolds' paired *F*_st_.

Distance between species and Reynolds' paired *F*_st_							
16S rRNA gene for the *Ara* genus							
*Ara* spp.	1	2	3	4	5	6	7
1. *A. severus*		0.978	1.000	0.985	0.934	1.000	0.790
2. *A. ararauna*	0.027		0.980	0.961	0.889	0.978	0.653
3. *A. rubrogenys*	0.041	0.025		0.954	0.500	1.000	0.636
4. *A. macao*	0.037	0.031	0.037		0.605	0.893	0.543
5. *A. chloropterus*	0.046	0.032	0.039	0.011		0.583	0.447
6. *A. militaris*	0.041	0.030	0.036	0.005	0.011		0.529
7. *A. ambiguus*	0.041	0.030	0.036	0.005	0.011	0.002	

*Note:* The lower diagonal contains the genetic distances between species, while the upper diagonal presents Reynolds' pairwise *F*_st_ values.

##### 3.1.3.3 Reynolds' Paired *F*_st_ of the Amazona and Ara Genera for the COI and 16S rRNA Genes

The results of pairwise genetic differentiation (Reynolds' paired *F*_st_) allowed identifying the species with the least genetic differentiation within each genus and marker. In the COI gene, the lowest genetic differentiation in the *Amazona* genus was observed between *Amazona ochrocephala* and *Amazona aestiva* (*F*_st_ = 0.9247) (Table 3), while in *Ara*, the pair with the least differentiation corresponded to *Ara militaris* and *Ara ambiguus* (*F*_st_ = 0.8333) (Table 5). In the 16S rRNA gene, the lowest genetic differentiation in the *Amazona* genus was found between *Amazona autumnalis* and *Amazona amazonica* (0.188) (Table 4); in this marker, some values could not be calculated because the species involved shared the same haplotype and were represented by a single individual of the species. On the other hand, in the *Ara* genus, the lowest differentiation was observed between *Ara chloropterus* and *Ara ambiguus* (*F*_st_ = 0.4474) (Table 6).

#### 3.1.4 Phylogenetic Analysis

##### 3.1.4.1 Phylogenetic Inference of the Amazona Genus for the COI and 16S rRNA Gene Fragments

**FIGURE 2:**
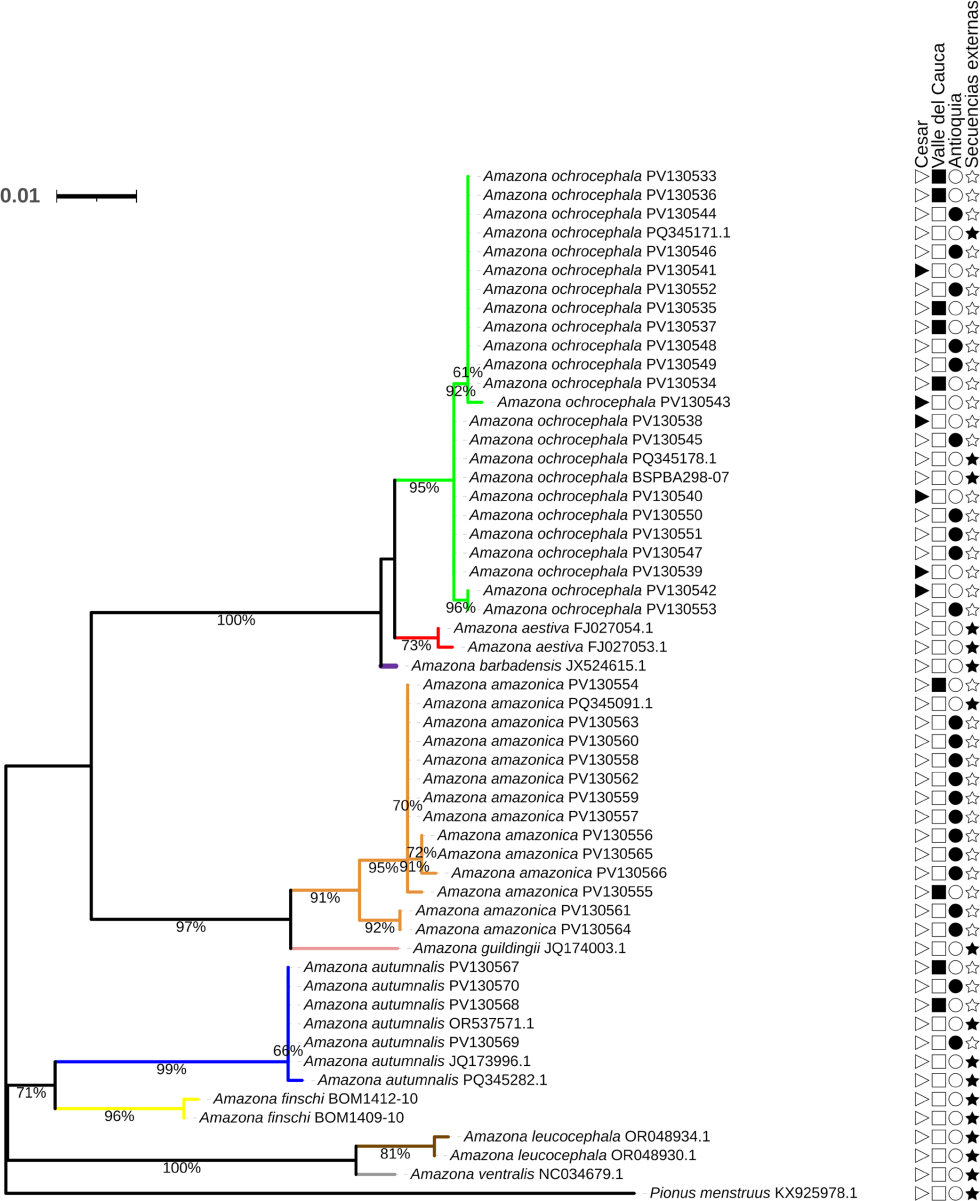
Phylogenetic tree of the *Amazona* genus from a fragment of the COI mitochondrial gene. Phylogenetic tree from fragments of the COI gene of the *Amazona* genus, generated by the Maximum Likelihood (ML) method using the HKY + F + G4 evolutionary model with a bootstrap of 1000 replicates. The analysis was based on 38 sequences from the current study and 17 partial sequences obtained from the NCBI and BOLD Systems. Branches are colored according to the species: lime green ( *Amazona ochrocephala* ), red ( *Amazona aestiva* ), purple ( *Amazona barbadensis* ), orange ( *Amazona amazonica* ), pink ( *Amazona guildingii* ), blue ( *Amazona autumnalis* ), yellow ( *Amazona finschi* ), brown ( *Amazona leucocephala* ), and gray ( *Amazona ventralis* ). A sequence from the species *Pionus menstruus* was included as an outgroup (“Secuencias externas”). The symbols on the right side indicate the origin of the sequences: a star (★) represents sequences from external databases (NCBI and BOLD Systems), a triangle (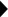) corresponds to individuals from the Cesar region, a square (▆) shows individuals from the Valle del Cauca region, and a circle (●) indicates individuals from the Antioquia region.

The phylogenetic analysis of the COI mitochondrial gene for the *Amazona* genus showed that all birds generally clustered with individuals of the same species (Figure 2). However, *Amazona aestiva* (red clade), *Amazona barbadensis* (purple clade), and *Amazona ochrocephala* (lime green clade) clustered within a major clade with 100% branch support, although each species was distributed in distinct subclades within this group. This pattern is consistent with genetic distances analyses (Table 3), where the lowest genetic distance was observed among these three species. On the other hand, the remaining species formed more differentiated clades, with branch support higher than 71%. Furthermore, this clustering is also supported by paired *F*_st_ values, which reflect less genetic differentiation among these species, possibly associated with more recent or continuous gene flow.

**FIGURE 3:**
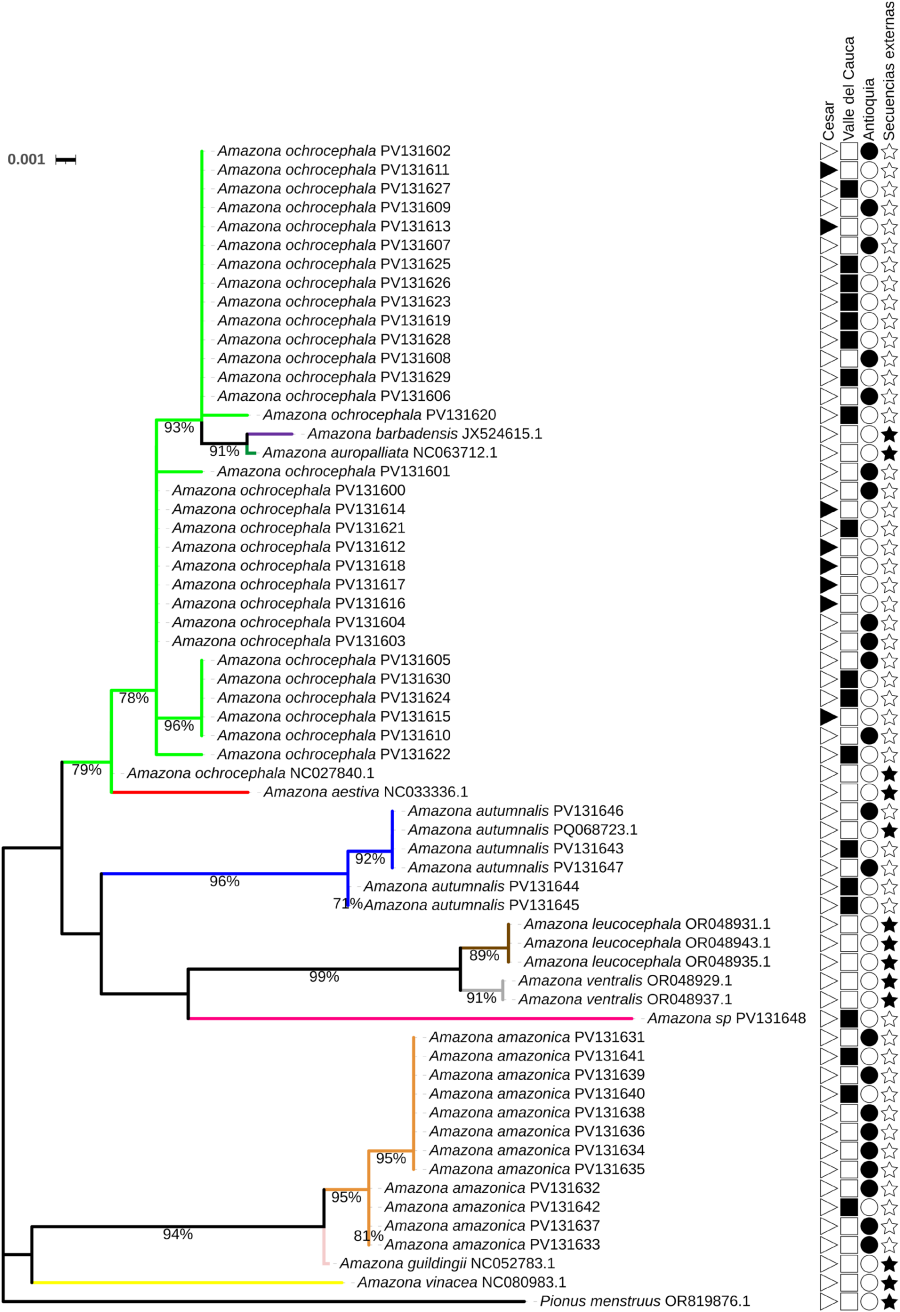
Phylogenetic tree of the *Amazona* genus from a fragment of the 16S rRNA mitochondrial gene. Phylogenetic tree from 16S rRNA gene fragments of the *Amazona* genus, generated by the Maximum Likelihood (ML) method using the HKY + F + I evolutionary model with a bootstrap of 1000 replicates. The analysis was based on 49 sequences from the current study and 13 partial sequences obtained from NCBI. Branches are colored according to the species: lime green ( *Amazona ochrocephala* ), purple ( *Amazona barbadensis* ), green ( *Amazona auropalliata* ), red ( *Amazona aestiva* ), blue ( *Amazona autumnalis* ), brown ( *Amazona leucocephala* ), gray ( *Amazona ventralis* ), fuchsia (*Amazona* sp.), orange ( *Amazona amazonica* ), pink ( *Amazona guildingii* ), and yellow ( *Amazona vinacea* ). A sequence from the species *Pionus menstruus* was included as an outgroup (“Secuencias externas”). The symbols on the right side indicate the origin of the sequences: a star (★) represents sequences from external databases, a triangle (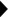) corresponds to individuals from the Cesar region, a square (▆) shows individuals from the Valle del Cauca region, and a circle (●) indicates individuals from the Antioquia region.

In the case of the 16S rRNA gene fragment, several species, including *Amazona ochrocephala* , *Amazona aestiva* , *Amazona barbadensis* , and *Amazona auropalliata* , were grouped within the same clade with 79% branch support (Figure 3). This result may be due to the low variability of the evaluated 16S rRNA gene fragment, which hinders the ability to delimit these species taxonomically, a pattern consistent with the low genetic distances previously observed (Table 4). Besides, these species have previously been reported to belong to a species complex, which explains their grouping in the phylogenetic tree (Eberhard and Bermingham 2004; Ribas et al. 2007; Urantówka et al. 2014). On the other hand, the other species were grouped into separate clades, formed exclusively by individuals of the same species, with branch support higher than 94%, indicating clear genetic differentiation among them. Finally, the individual reported as *Amazona* sp. was positioned closer to the clades corresponding to *Amazona leucocephala* and *Amazona ventralis* . This individual was named *Amazona* sp. because it could not be morphologically identified to the species level.

##### 3.1.4.2 Phylogenetic Inference of the Ara Genus for the COI and 16S rRNA Genes

**FIGURE 4:**
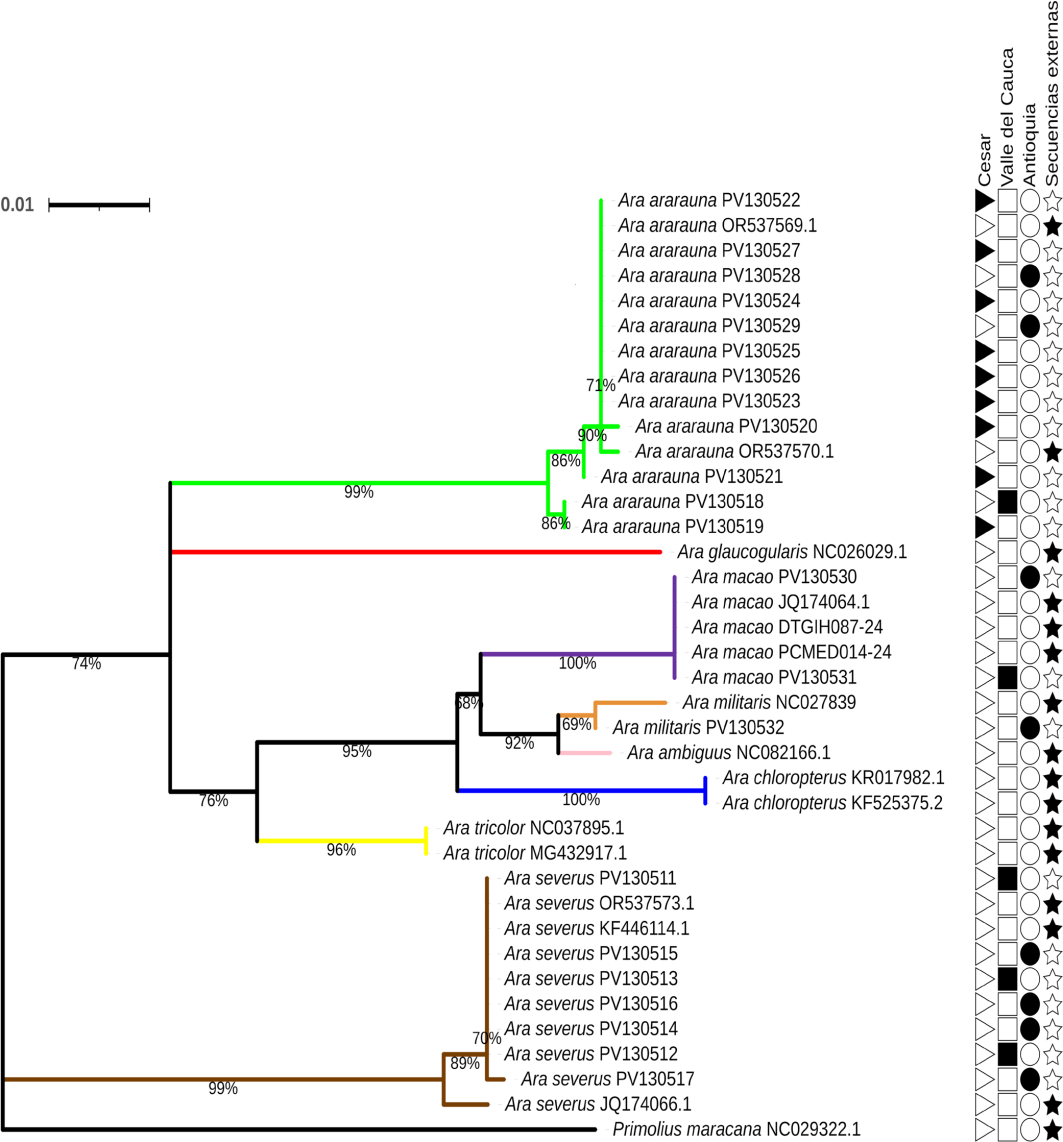
Phylogenetic tree of the *Ara* genus from a fragment of the COI mitochondrial gene. Phylogenetic tree from fragments of the COI gene of the *Ara* genus, generated by the Maximum Likelihood (ML) method using the TN + F + G4 evolutionary model with a bootstrap of 1000 replicates. The analysis was based on 22 sequences from the current study and 16 partial sequences obtained from NCBI and BOLD Systems. The branches are colored according to the species: lime green ( *Ara ararauna* ), red ( *Ara glaucogularis* ), purple ( *Ara macao* ), orange ( *Ara militaris* ), pink ( *Ara ambiguus* ), blue ( *Ara chloropterus* ), yellow ( *Ara tricolor* ), and brown ( *Ara severus* ). A sequence from the species *Primolius maracana* was included as an outgroup (“Secuencias externas”). The symbols on the right side indicate the origin of the sequences: a star (★) represents sequences from external databases, a triangle (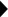) corresponds to individuals from the Cesar region, a square (▆) shows individuals from the Valle del Cauca region, and a circle (●) indicates individuals from the Antioquia region.

For the *Ara* genus, phylogenetic analysis of the COI gene showed that *Ara severus* grouped in an independent clade, separate from the rest of the species. This separation is consistent with the results of the genetic distances (Table 5), which indicate a greater divergence of *Ara severus* compared to the other species. On the other hand, *Ara ambiguus* and *Ara militaris* grouped closely together, with a branch support of 92%. The other species formed clades, with branch support values higher than 96%, indicating a clear genetic differentiation between them (Figure 4).

**FIGURE 5:**
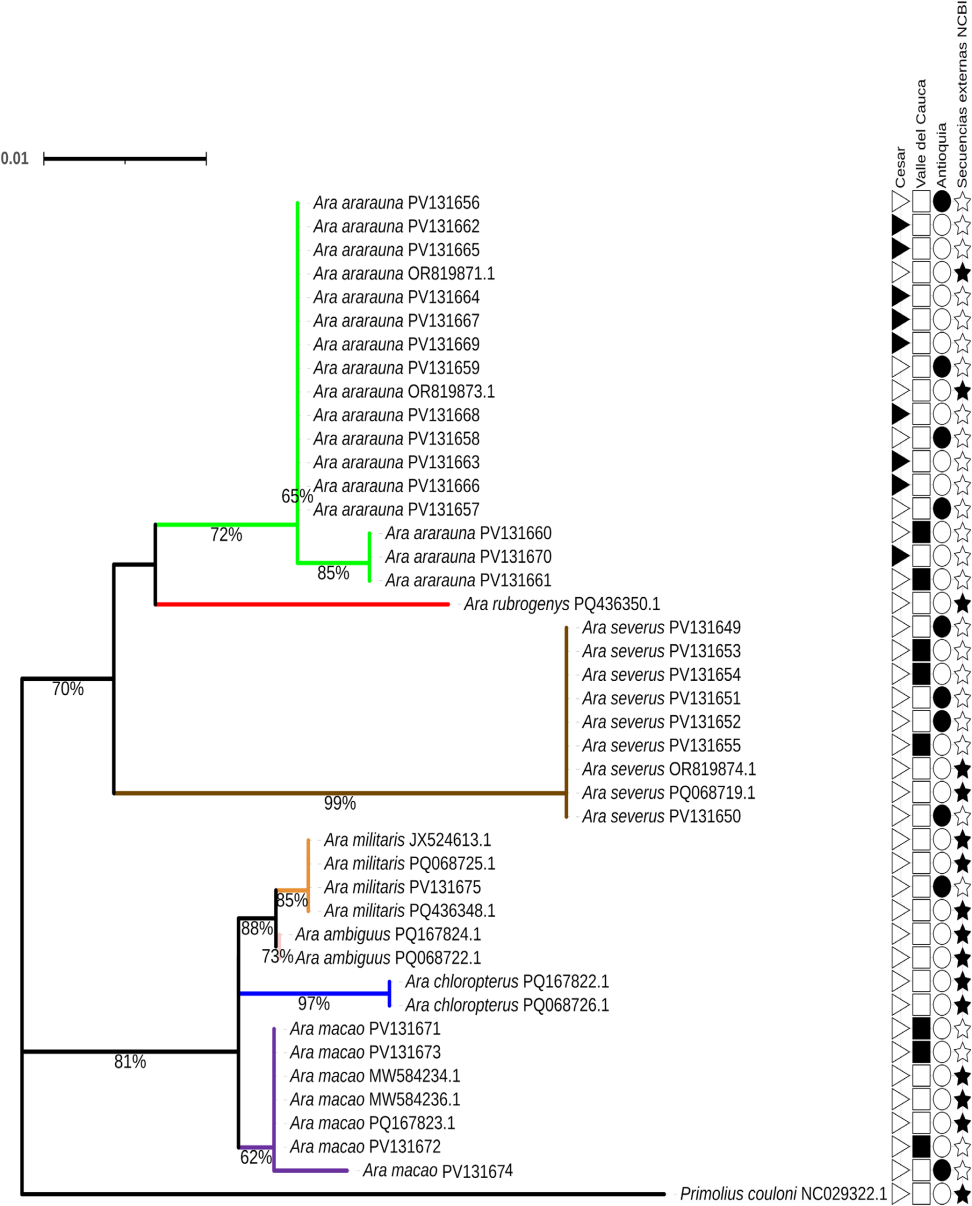
Phylogenetic tree of the *Ara* genus from a fragment of the 16S rRNA mitochondrial gene. Phylogenetic tree based on the 16S rRNA gene of the *Ara* genus, generated by the Maximum Likelihood (ML) method using the HKY + F + I evolutionary model with a bootstrap of 1000 replicates. The analysis was based on 27 sequences from the current study and 16 partial sequences obtained from NCBI. Branches are colored according to species: lime green ( *Ara ararauna* ), red ( *Ara rubrogenys* ), brown ( *Ara severus* ), orange ( *Ara militaris* ), pink ( *Ara ambiguus* ), blue ( *Ara chloropterus* ), and purple ( *Ara macao* ). A sequence from the species *Primolius maracana* was included as an outgroup (“Secuencias externas”). The symbols on the right side indicate the origin of the sequences: a star (★) represents sequences from external databases, a triangle (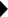) corresponds to individuals from the Cesar region, a square (▆) shows individuals from the Valle del Cauca region, and a circle (●) indicates individuals from the Antioquia region.

In the case of the evaluated fragment of the 16S rRNA gene, phylogenetic analysis revealed the formation of two main clades (Figure 5). The first clade grouped the species *Ara ararauna* , *Ara rubrogenys* , and *Ara severus* , while the second included *Ara militaris* , *Ara ambiguus* , *Ara chloropterus* , and *Ara macao* . Within each subclade, all species grouped exclusively with individuals of their own species, with branch support > 70%.

The most distantly related species on the phylogenetic tree were *Ara macao* and *Ara ararauna* . This result differs from the genetic distances analyses, which identified *Ara chloropterus* and *Ara severus* as the species with the greatest genetic differences between them (Table 6). On the other hand, the closest related species were *Ara ambiguus* and *Ara militaris* , which are consistent with the distances analyses, which showed that these species had the lowest divergence values.

#### 3.1.5 Haplotype Networks and Principal Coordinates Analysis

##### 3.1.5.1 Genetic Variability Analysis for the Amazona Genus Using the COI and 16S Genes

**FIGURE 6:**
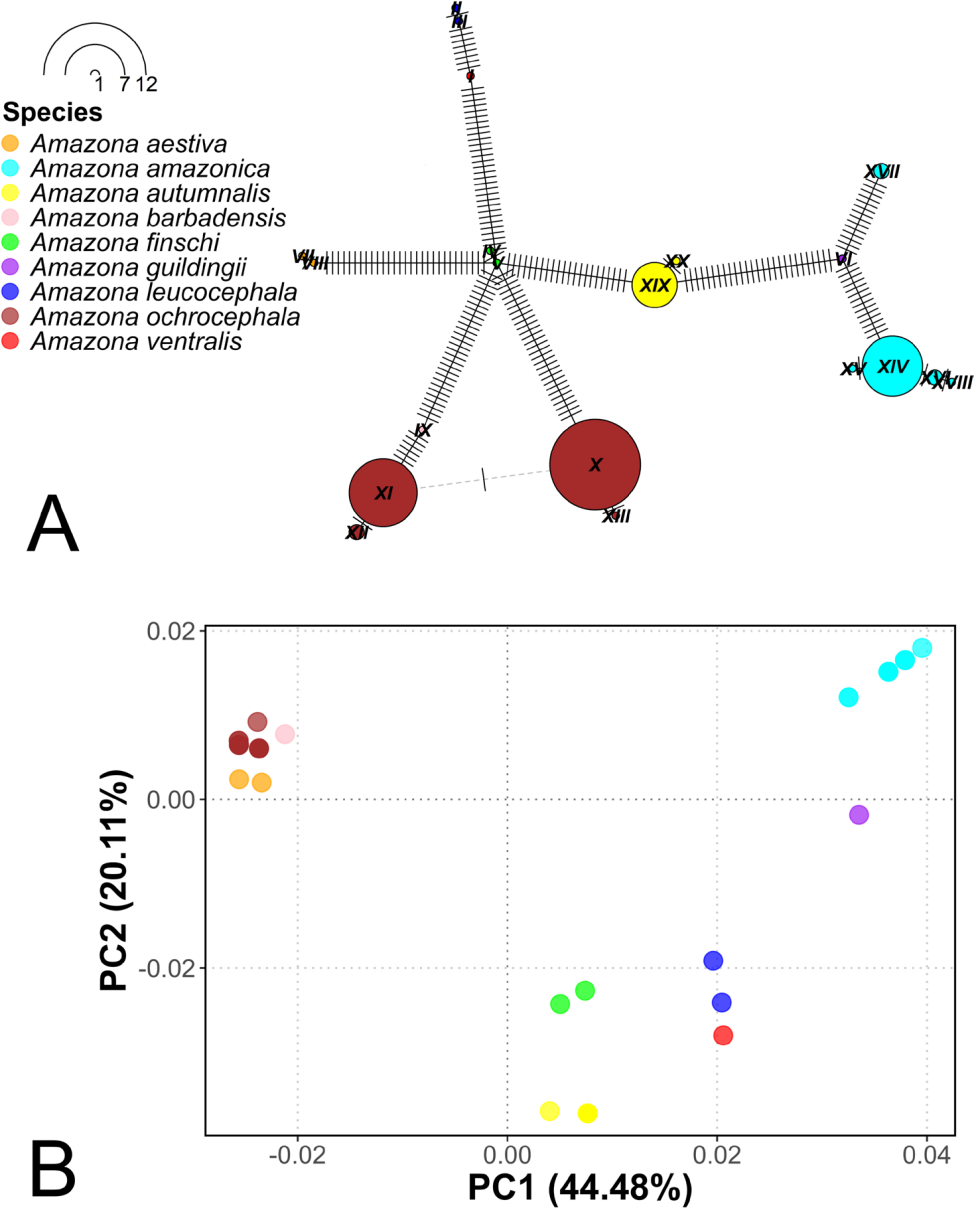
Haplotype network and principal coordinates analysis (PCoA) based on the COI mitochondrial gene for the *Amazona* genus. (A) Haplotype network based on a fragment of the COI mitochondrial gene for the *Amazona* genus. The size of the nodes is proportional to the frequency of each haplotype, and the lines represent the number of mutations between haplotypes. (B) Principal coordinates analysis (PCoA) showing the genetic clustering among species of the *Amazona* genus.

The haplotype network analysis based on the evaluated fragment of the COI gene for the *Amazona* genus identified a total of 20 haplotypes. Each species presented unique haplotypes, that is, no haplotypes were shared between the different species. *Amazona aestiva* , *Amazona ochrocephala* , and *Amazona barbadensis* share a common ancestor, which is consistent with their grouping in the same clade according to phylogenetic analyses (Figure 2) and their coincidence in the same quadrant of the PCoA. Furthermore, *Amazona ochrocephala* and *Amazona barbadensis* showed the lowest number of mutations among their haplotypes (six mutations), indicating a closer genetic relationship. This pattern is consistent with the results of the phylogenetic tree (Figure 2), the genetic distances analysis (Table 3), and the PCoA (Figure 6B), suggesting a recent evolutionary divergence between these two species.

The PCoA based on genetic distances reinforces the findings observed in the haplotype network (Figure 6A). This analysis explained 44.48% and 20.11% of the genetic variation in the PC1 and PC2 components, respectively, allowing the visualization of the genetic differentiation between species of the *Amazona* genus. Species such as *Amazona ochrocephala* and *Amazona ventralis* showed greater dispersion in the plane, indicating a higher genetic diversity among these species, as observed in the genetic distances analysis (Table 3). On the other hand, the close clustering of *Amazona aestiva* , *Amazona ochrocephala* , and *Amazona barbadensis* in the PCoA space coincides with their genetic relationships observed in the haplotype network and phylogenetic analysis.

**FIGURE 7:**
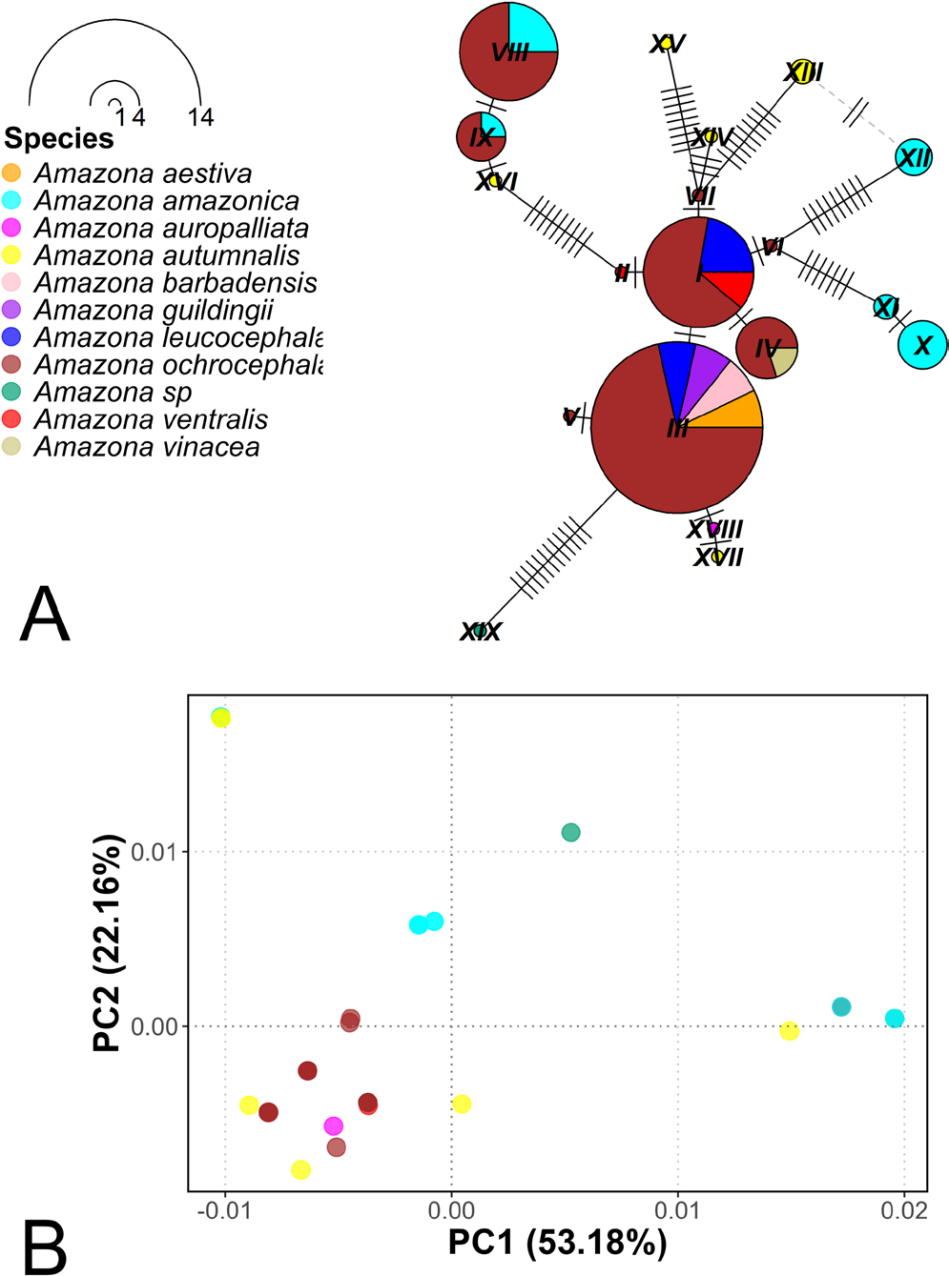
Haplotype network and principal coordinates analysis (PCoA) based on the 16S rRNA mitochondrial gene for the *Amazona* genus. (A) Haplotype network based on the evaluated fragment of the 16S rRNA mitochondrial gene for *Amazona* spp., where the size of the nodes is proportional to the frequency of each haplotype and the lines represent the number of mutations. (B) Principal coordinates analysis (PCoA) showing the genetic clustering among species of the *Amazona* genus.

In the case of the 16S rRNA gene fragment, a total of 19 haplotypes were identified (Figure 7A). Contrary to what was observed with the COI gene, most species did not present unique haplotypes, that is, haplotypes were shared between different species. The only exception was the species *Amazona auropalliata* , which presented a single haplotype. This pattern suggests that the 16S rRNA gene, under this type of analysis, is not adequate to discriminate the *Amazona* genus at the taxonomic species level, since it does not allow a clear differentiation between species. The PCoA presents findings similar to those observed in the haplotype network (Figure 7B). This analysis explained 53.18% and 22.16% of the genetic variation in components PC1 and PC2, respectively. Unlike the analysis with the COI gene, the PCoA shows greater overlap between species of the *Amazona* genus, reflecting the lack of genetic differentiation between them with this gene, reinforcing the idea that this genetic marker is not suitable for distinguishing species within this genus.

##### 3.1.5.2 Genetic Variability Analysis for the Ara Genus Using the COI and 16S rRNA Genes

**FIGURE 8:**
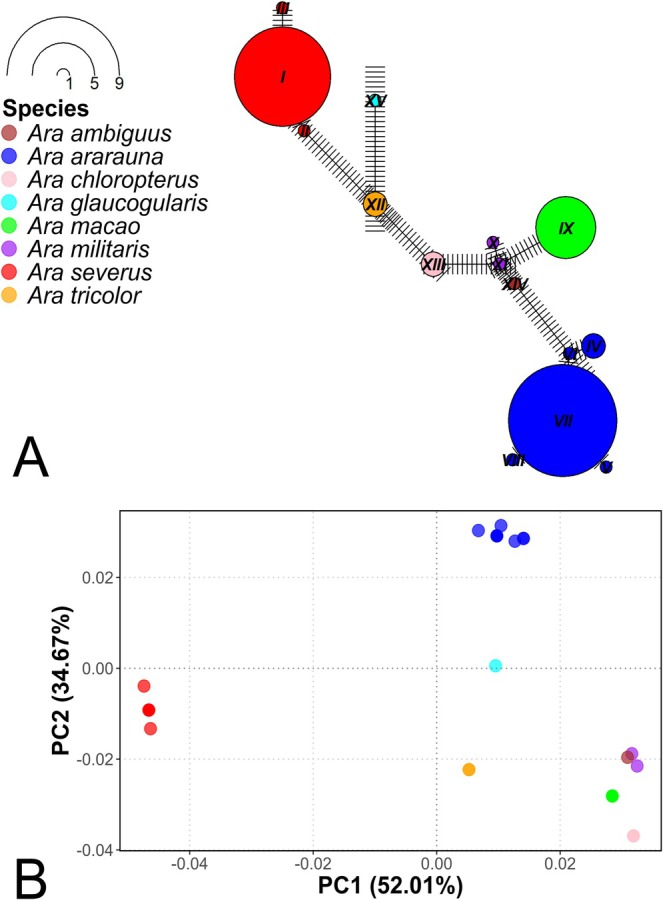
Haplotype network and principal coordinates analysis (PCoA) based on a fragment of the COI mitochondrial gene for the *Ara* genus. (A) Haplotype network based on a fragment of the COI mitochondrial gene for the *Ara* genus, where the size of the nodes is proportional to the frequency of each haplotype and the lines represent the number of mutations. (B) Principal coordinates analysis (PCoA) showing the genetic clustering among species of the *Ara* genus.

Haplotype network analysis based on the evaluated fragment of the COI gene for the *Ara* genus identified a total of 15 haplotypes. Each species presented unique haplotypes, that is, no haplotypes were shared between the different species, but some species presented two or more haplotypes, such as *Ara ararauna* , which presented haplotypes IV, V, VI, VII, and VIII (Figure 8A). The most genetically related species were *Ara militaris* and *Ara ambiguus* , with the lowest number of mutations among all species (six mutations) (Figure 8A). This pattern is consistent with the results of the phylogenetic tree (Figure 4), the genetic distances analysis (Table 5), and the PCoA (Figure 8B).

The PCoA explained 52.01% and 34.67% of the genetic variation along the PC1 and PC2 axes, respectively, allowing the visualization of the genetic differentiation among most species of the *Ara* genus (Figure 8B). However, there was an overlap between individuals of the species *Ara militaris* and *Ara ambiguus* , indicating that these species could be the same or closely related. *Ara severus* was the species with the greatest separation on the plane, suggesting that this species has the highest genetic diversity compared to the other species, coinciding with the analysis of genetic distances (Table 5), the phylogenetic tree (Figure 4), and the haplotype network (Figure 8A).

**FIGURE 9:**
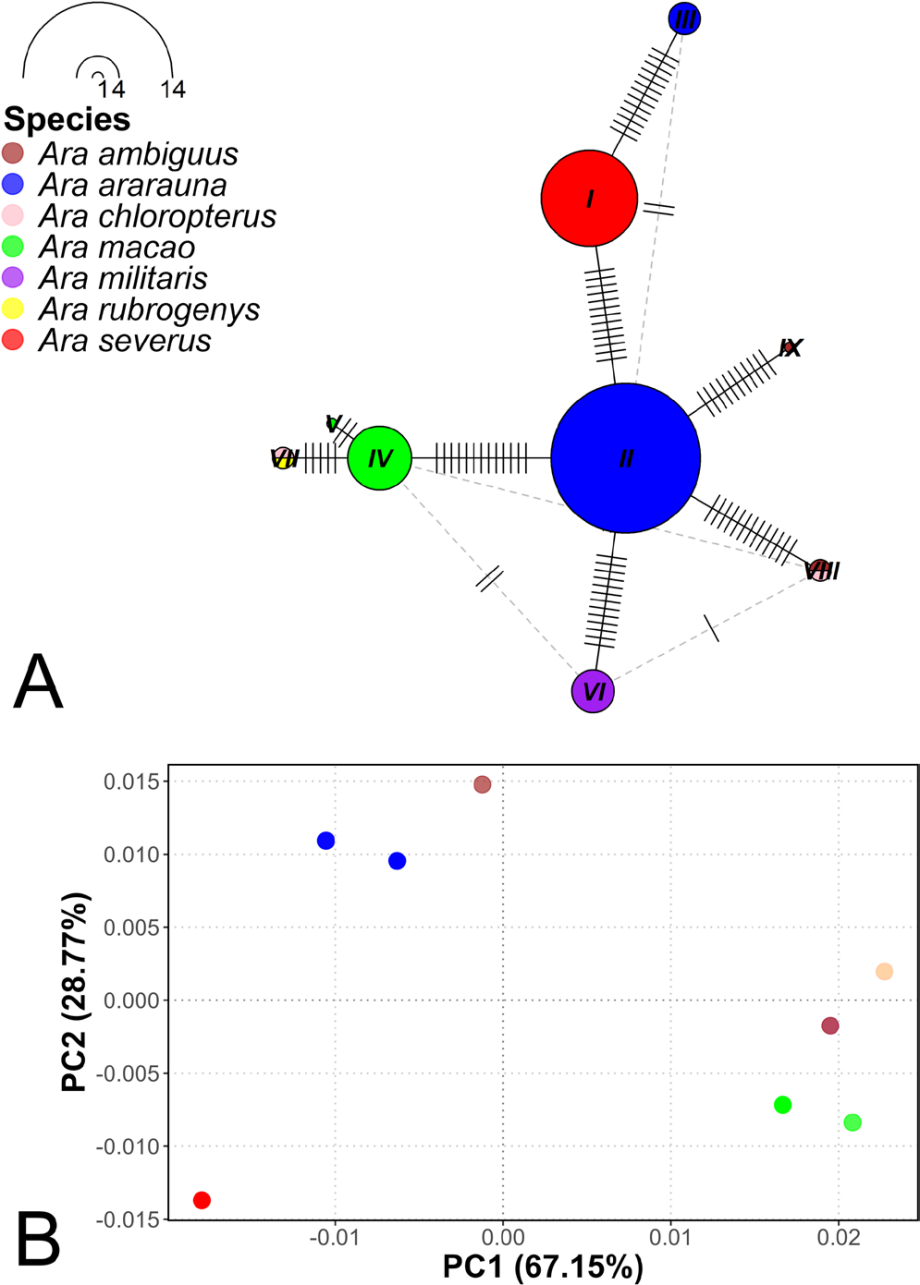
Haplotype network and principal coordinates analysis (PCoA) based on the 16S rRNA mitochondrial gene for the *Ara* genus. (A) Haplotype network based on the 16S rRNA mitochondrial gene for the *Ara* genus, where the size of the nodes is proportional to the frequency of each haplotype and the lines represent the number of mutations. (B) Principal coordinates analysis (PCoA) showing the genetic clustering among species of the *Ara* genus.

The haplotype network analysis based on a 16S rRNA gene fragment for the *Ara* genus identified a total of nine haplotypes (Figure 9A). While *Ara macao* , *Ara ararauna* , and *Ara militaris* presented unique haplotypes, *Ara chloropterus* shared haplotypes with *Ara rubrogenys* and *Ara ambiguus* . This pattern suggests that the 16S rRNA gene has a limited ability to discriminate between species within the *Ara* genus compared to the COI gene due to the presence of shared haplotypes among different species.

The 16S rRNA gene‐based PCoA reinforces the findings observed in the haplotype network (Figure 9B). This analysis explained 67.15% and 28.77% of the genetic distance on the PC1 and PC2 axes, respectively. The species *Ara severus* shows the greatest separation from the other species, suggesting higher genetic diversity within this species in the *Ara* genus. On the other hand, *Ara rubrogenys* overlaps with *Ara chloropterus* , while *Ara militaris* also shows overlap with *Ara chloropterus* in the PCoA.

## 4 Discussion

The implementation of methodologies such as DNA barcoding in birds that are victims of illegal trafficking is an essential tool for addressing challenges related to species identification and the assessment of their genetic diversity (Gonçalves et al. 2015). In this study, the use of mitochondrial markers such as fragments of the COI and 16S rRNA genes allowed the identification of biological material at the species level in individuals of the *Ara* and *Amazona* genera confiscated in Colombia. The COI and 16S rRNA mitochondrial genes proved valuable for species identification in the *Amazona* and *Ara* genera, supporting species‐level differentiation under mitochondrial markers, although with differences in their ability to delimit species. This study allowed the identification of the species complex known as yellow‐headed parrots, which includes *Amazona ochrocephala* , *Amazona aestiva* , *Amazona barbadensis* , and *Amazona auropalliata*. This group, as other authors have pointed out, is characterized by its close genetic relationship due to a recent evolutionary divergence, which makes its molecular identification at the species level difficult (Arias‐Sosa et al. 2024; Eberhard and Bermingham 2004; Ribas et al. 2007). This genetic closeness between some species explains the limitations observed in certain mitochondrial markers, such as the 16S rRNA gene, for discriminating between them. In contrast, the COI gene was able to differentiate the evaluated species within the *Amazona* genus, since unique haplotypes were identified, supporting its effectiveness for taxonomic identification within this group of birds.

In this study, the COI gene showed a better performance compared to 16S rRNA; this result is supported by the genetic diversity indices and the statistics of the obtained sequences (Table 2 and Table S3), where the *F*_st_ values for the COI gene were higher compared to 16S rRNA (*Amazona* spp.: COI = 0.9608, 16S rRNA = 0.0017; *Ara* spp.: COI = 0.9630, 16S rRNA = 0.7455). This indicates a greater genetic differentiation between species of both genera when COI is used. The *H*_s_ in COI was considerably lower (*Amazona* spp.: *H*_s_ = 0.0128, *Ara* spp.: *H*_s_ = 0.0113) than in 16S rRNA (*Amazona* spp.: *H*_s_ = 0.1663, *Ara* spp.: *H*_s_ = 0.0767). Additionally, COI presented a higher number of variable sites compared to 16S rRNA, suggesting that COI has a greater capacity to detect differences between species, while 16S rRNA is a more conserved gene with lower variability rates within the analyzed groups.

These results are consistent with previous evidence indicating that 16S rRNA is a more conserved marker and, therefore, its application is more useful at higher taxonomic levels, while COI allows a better resolution at the species level as it has a higher variability rate (Goyal and Sobti 2022; De Mandal et al. 2014). Furthermore, at the specific level, variable genetic diversity was observed between species of both genera. For example, in the 16S rRNA gene, *Amazona ochrocephala* and *Amazona amazonica* presented *H*_s_ values of 0.136 and 0.233, respectively, while in COI, these same species recorded *H*_s_ values of 0.009 and 0.027. In the *Ara* genus, *Ara severus* and *Ara ararauna* showed low but distinct values between genes; with the 16S rRNA gene, *H*_s_ values were 0 and 0.018, respectively, and with the COI gene, *H*_s_ values were 0.013 and 0.015, respectively. These results show that although COI showed greater interspecific resolution, in species with some intraspecific variability, such as *Amazona amazonica* , *Amazona ochrocephala* , *Ara ararauna* , and *Ara severus* , these markers could be valuable for population connectivity studies, gene flow assessment, or reintroduction processes at a regional scale.

The different analyses carried out in this study showed that the COI gene is more suitable for the molecular identification of species belonging to the *Amazona* and *Ara* genera, since most species grouped clearly with individuals of the same species in the phylogenetic analyses (Figures 2 and 4), haplotype networks (Figures 6A and 8A), and PCoA (Figures 6B and 8B). In these analyses, each species presented unique haplotypes. Moreover, in the PCoA, a clear differentiation between species was observed, except for the yellow‐headed parrot species complex, constituted by *Amazona aestiva* , *Amazona ochrocephala* , and *Amazona barbadensis* , where the species grouped closely together. Likewise, within the *Ara* genus, low genetic diversity was observed between *Ara militaris* and *Ara ambiguus* , reflected in a lower genetic distance (0.010). This result is reflected in a recent coalescence in the phylogenetic analyses (Figure 4), as well as in the haplotype networks, where a reduced number of mutations is evident, separating the haplotypes of each of these species (Figure 8A). Furthermore, in the PCoA analysis (Figure 8B), a partial overlap was observed between these two species, so the haplotype analysis offers a better genetic resolution in these cases, as evidenced by the presence of specific haplotypes for each of them (Figure 8A). However, all the analyses supported species‐level differentiation between *Ara militaris* and *Ara ambiguus* under mitochondrial markers, although these species are closely related as reported by Eberhard et al. (2015). These results are consistent with previous studies that have shown that the COI gene is a highly effective marker for species delimitation (Dimitrioud et al. 2017; Hebert et al. 2004; Nougoue 2012; Yang et al. 2010). Different authors indicate that this gene is one of the most effective markers for the identification of different bird species due to its variability rate, which makes it an effective tool for molecular identification (Mendoza et al. 2016; Pulgarín‐R et al. 2021).

On the other hand, the 16S rRNA gene showed important limitations in taxonomic discrimination at the species level. Haplotype networks and principal components analyses showed overlap between species and shared haplotypes, suggesting a lower capacity to resolve recent evolutionary relationships. This is because the 16S rRNA gene is a highly conserved gene with a lower mutation rate, where its low variability reduces the number of variable and parsimoniously informative sites, limiting its ability to detect differences between closely related species (Table 2). This pattern is observed in haplotype networks (Figures 7A and 9A), where several species share haplotypes, and in the PCoA (Figures 7B and 9B), where greater overlap between different species is observed. Previous research supports these findings, showing that this gene, although valuable for studies at higher taxonomic levels such as family and genus, is not effective for discriminating at the species level due to its lower genetic variability (Goyal and Sobti 2022; Wang et al. 2018; Xia et al. 2012).

In addition to their usefulness for taxonomic identification, mitochondrial markers, particularly the COI gene, have been used as tools to define the probable origin of parrots confiscated from illegal trafficking in Colombia. Arias‐Sosa et al. (2024) used this gene to identify the phylogeographic structure of several species of the *Amazona* genus and guide responsible release processes. In the current work, PCoA analyses based on the COI gene showed clear genetic differentiation between some *Amazona* and *Ara* species (Figures 6B and 8B). In particular, in *Amazona amazonica* , greater dispersion of the points in the PCoA was observed, along with a higher number of haplotypes within this same species, indicating the existence of genetic differentiation between populations and, therefore, the possibility of using this gene to study their population structuring. Similarly, *Ara severus* and *Ara ararauna* also showed dispersion in the PCoA, suggesting a certain degree of genetic variability within these species for this gene. However, in the case of *Ara severus* and *Ara ararauna* , the lack of sequences from wild populations from Colombia in global databases prevents provenance studies on individuals recovered from trafficking. *Amazona amazonica* , on the other hand, does have representative sequences from different Colombian wild populations, which would allow determining the possible origin of confiscated individuals and carrying out their appropriate reintroduction. This approach is crucial to prevent the introduction of individuals into genetically distinct populations, which could lead to genetic erosion or the alteration of local population dynamics (Carmona and Arango 2011).

Finally, the results obtained in this study could be complemented by the use of nuclear markers, such as microsatellites or single sequence repeats (SSRs) and single‐nucleotide polymorphisms (SNPs). Since mitochondrial markers reflect only maternal inheritance, the incorporation of nuclear markers, which are inherited from both parents, provides a more comprehensive view of the evolutionary history and phylogenetic relationships of species (Hill 2017; Welch et al. 2011). SSRs have been widely used in genetic diversity and population structuring studies due to their high variability and ability to detect changes at the intraspecific level (Escalante‐Pliego et al. 2022; Willows‐Munro and Kleinhans 2020). On the other hand, SNPs allow for more detailed genomic inferences and have proven to be valuable tools in the genetic structuring of wild populations, the identification of species and hybrids, as well as in the detection of introgression and hybridization events and traces of selection (Agazzi Migotto et al. 2023; Capel et al. 2022; Gautschi et al. 2024).

This study demonstrated that DNA barcoding, particularly the COI mitochondrial gene, is an effective tool for the taxonomic identification of parrots and macaws recovered from illegal trafficking in Colombia. The results indicated that the COI gene allows for more precise species delimitation, revealing unique haplotypes and clear differentiation in phylogenetic and principal components analyses. On the other hand, the 16S rRNA gene showed limitations in taxonomic discrimination due to its low genetic variability, which generated overlaps between species and hindered their differentiation. However, these markers could be used in future studies focused on species reintroduction processes or population structuring studies, especially if applied to more diverse groups with a higher number of haplotypes.

For the *Amazona* genus, the COI gene allowed a clear separation between most species, with unique haplotypes and well‐defined clusters in phylogenetic and haplotype network analyses. However, in the yellow‐headed parrot complex ( *Amazona ochrocephala* , *Amazona aestiva* , and *Amazona barbadensis* ), less genetic differentiation was observed, suggesting recent divergence or gene flow. In contrast, the 16S rRNA gene showed poorly defined clusters and shared haplotypes between species, confirming its lower resolution in this genus. The inclusion of newly generated COI and 16S rRNA sequences from confiscated individuals contributes valuable reference data for this taxonomically complex group, improving identification reliability in global databases such as BOLD and GenBank.

For the *Ara* genus, the COI gene performed well in taxonomic resolution, allowing clear differentiation between most of the analyzed species. However, *Ara militaris* and *Ara ambiguus* showed high genetic similarity, reflected in their close grouping in phylogenetic analyses and some overlap in the PCoA, suggesting recent evolutionary differentiation. However, the combination of all analyses supports species‐level differentiation within this genus based on mitochondrial sequences. In contrast, the 16S rRNA gene showed similar limitations to those observed in the *Amazona* genus, with a lower capacity for species discrimination.

Future research should expand molecular characterization studies of Psittacidae using the COI gene, incorporating wild populations of species that showed higher numbers of haplotypes and greater intraspecific genetic diversity, such as *Amazona amazonica* , *Ara severus* , and *Ara ararauna* . Generating baseline genetic information from different regions of Colombia will allow the evaluation of potential population structuring according to geographic origin, which could help guide the reintroduction of confiscated individuals into their natural populations. In addition, it is essential to strengthen the generation, systematization, and publication of genetic reference data for Colombian Psittacidae species in databases such as GenBank and BOLD Systems. Enhancing the availability and quality of these records will improve molecular identification processes, support traceability studies, and facilitate the identification of derivatives and subproducts in illegal wildlife trade contexts.

## Author Contributions

**Julián Marín‐Villa:** conceptualization (equal), data curation (equal), formal analysis (equal), investigation (equal), methodology (equal), visualization (equal), writing – original draft (equal), writing – review and editing (equal). **Juan Carlos Rincón‐Flórez:** conceptualization (equal), data curation (equal), formal analysis (equal), writing – original draft (equal), writing – review and editing (equal). **Cristina Úsuga‐Monroy:** conceptualization (equal), formal analysis (equal), investigation (equal), methodology (equal), writing – original draft (equal), writing – review and editing (equal). **Albeiro López‐Herrera:** conceptualization (equal), formal analysis (equal), funding acquisition (lead), writing – original draft (equal), writing – review and editing (equal).

## Funding

This work was supported by Universidad Nacional de Colombia, HERMES 57690.

## Conflicts of Interest

The authors declare no conflicts of interest.

## Supporting information


**Figure S1:** PCR results in agarose gel of a COI gene fragment from individuals of the *Amazona* and *Ara* genera.
**Figure S2:** PCR results in agarose gel of a 16S rRNA gene fragment from individuals of the *Amazona* and *Ara* genera.
**Table S1:** External COI reference sequences used in phylogenetic and barcoding analyses.
**Table S2:** External 16S rRNA reference sequences used in phylogenetic and barcoding analyses.
**Table S3:** Genetic differentiation and diversity indices estimated from COI and 16S rRNA sequences in individuals of the genera *Amazona* and *Ara*.
**Table S4:** Specimen information and GenBank and BOLD accession numbers for COI sequences generated in this study.
**Table S5:** Specimen information and GenBank accession numbers for 16S rRNA sequences generated in this study.

## Data Availability

The data and DNA sequences obtained from each individual were deposited in the National Center for Biotechnology Information (NCBI) and Barcode of Life Data Systems (BOLD Systems) databases. The dataset associated with this study is available in BOLD Systems at: https://bench.boldsystems.org/index.php/MAS_Management_DataConsole?codes=UNCUR. Detailed accession numbers for all individuals are provided in Tables S4 and S5. Table S4 lists the COI gene accession numbers in GenBank and BOLD Systems, while Table S5 includes the 16S rRNA gene accession numbers in GenBank.
