# Supplementary material for: Mitochondrial Markers COI and 16S rRNA for the Molecular Identification of Parrots and Macaws Recovered From Illegal Trafficking in Three Areas of Colombia
Source: Ecol Evol. 2026 Mar 25;16(4):e73335. doi: 10.1002/ece3.73335 (PMC13107283; doi:10.1002/ece3.73335)
Supplement: Supplementary file 1 — Figure S1: PCR results in agarose gel of a COI gene fragment from individuals of the Amazona and Ara genera. Figure S2: PCR results in agarose gel of a 16S rRNA gene fragment from individuals of the Amazona and Ara genera. Table S1: External COI reference sequences used in phylogenetic and barcoding analyses. Table S2: External 16S rRNA reference sequences used in phylogenetic and barcoding analyses. Table S3: Genetic differentiation and diversity indices estimated from COI and 16S rRNA sequences in individuals of the genera Amazona and Ara. Table S4: Specimen information and GenBank and BOLD accession numbers for COI sequences generated in this study. Table S5: Specimen information and GenBank accession numbers for 16S rRNA sequences generated in this study. [file ECE3-16-e73335-s001.docx]

**Supplementary Figure S1:** PCR results in agarose gel of a COI gene fragment from individuals of the *Amazona* and *Ara* genera*.*


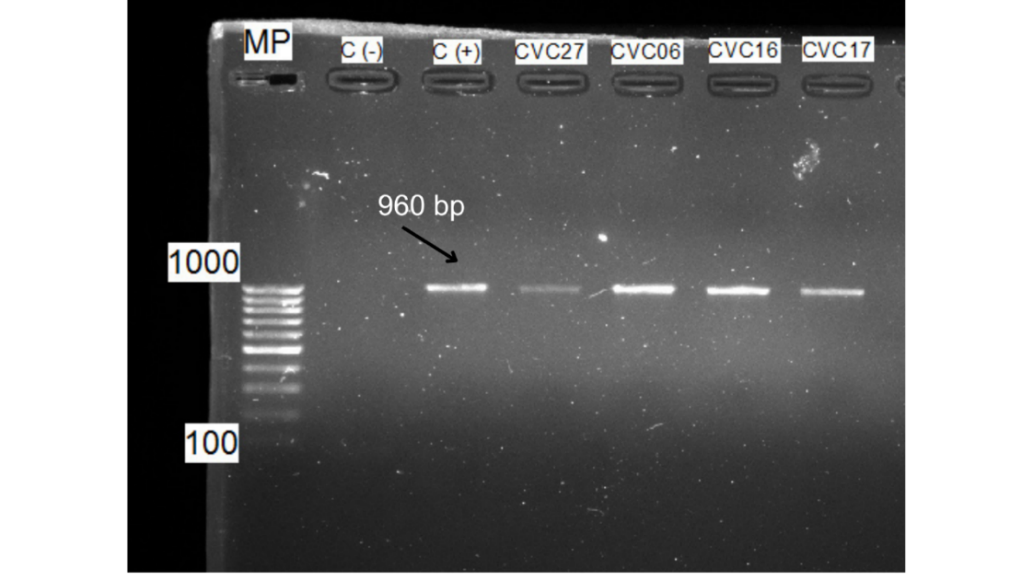


*1.2% agarose gel electrophoresis results of PCR amplicons of a COI gene fragment with a band size of 960 bp. Lane 1: 1000 bp molecular weight (MP) marker. Lane 2: Negative control (C -) with molecular-grade water. Lane 3: Positive control (C +) of DNA from a previously identified Psittacidae individual. Lane 4: Psittacidae sample identified as CVC27. Lane 5: Psittacidae sample identified as CVC06. Lane 6: Psittacidae sample identified as CVC16. Lane 7: Psittacidae sample identified as CVC17.*

**Supplementary Figure S2:** PCR results in agarose gel of a 16S rRNA gene fragment from individuals of the *Amazona* and *Ara* genera.


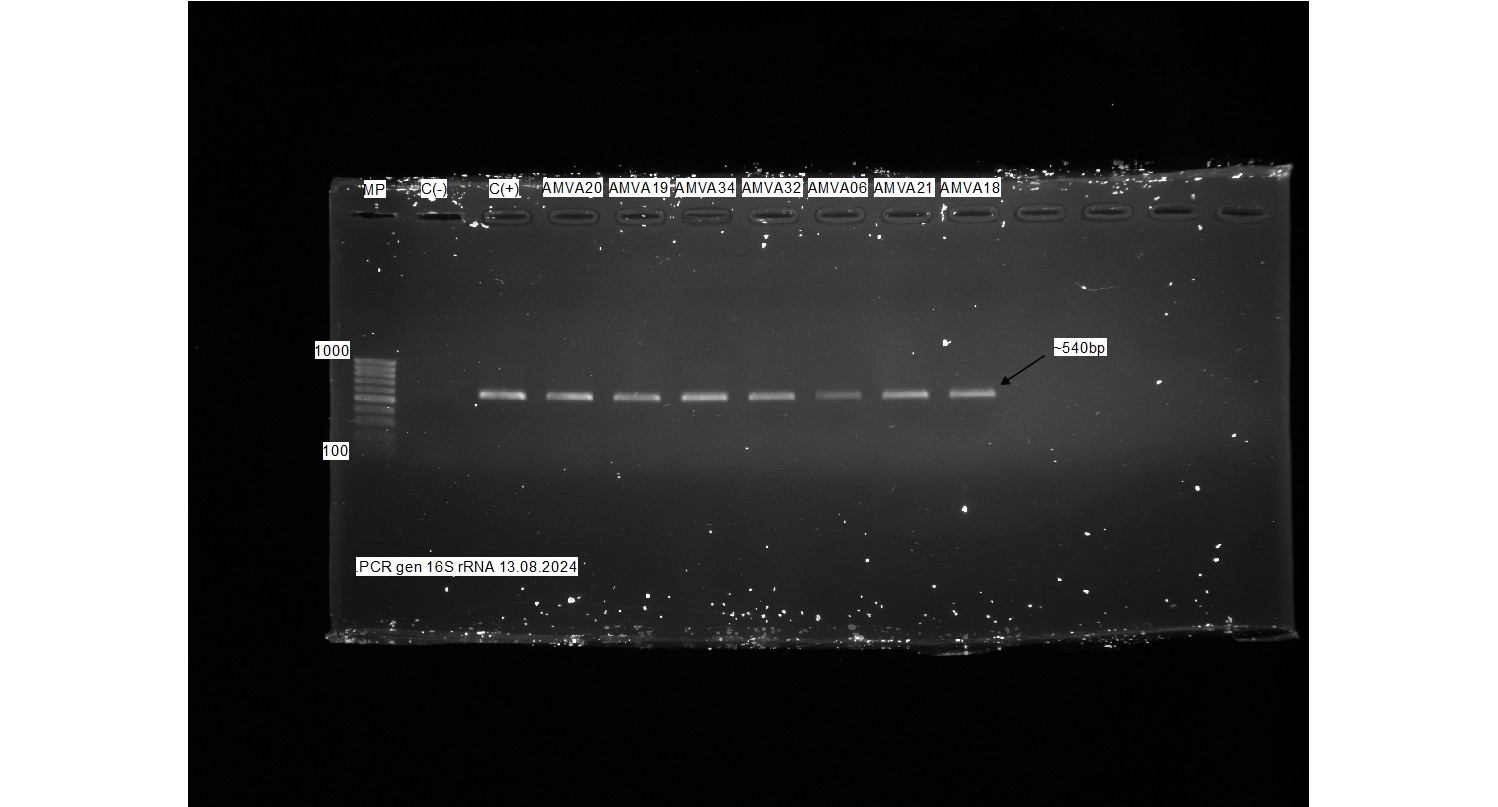


*1.2% agarose gel electrophoresis results of PCR amplicons of a 16S rRNA gene fragment with a band size of 540 bp. Lane 1: 1000 bp molecular weight (MP) marker. Lane 2: Negative control (C -) with molecular-grade water. Lane 3: Positive control (C +) of DNA from a previously identified Psittacidae individual. Lane 4: Psittacidae sample identified as AMVA20. Lane 5: Psittacidae sample identified as AMVA19. Lane 6: Psittacidae sample identified as AMVA34. Lane 7: Psittacidae sample identified as AMVA32. Lane 8: Psittacidae sample identified as AMVA06. Lane 9: Psittacidae sample identified as AMVA21. Lane 10: Psittacidae sample identified as AMVA18.*

**Supplementary Table S1**. External COI reference sequences used in phylogenetic and barcoding analyses.

| **Genus** | **Species** | **Accession number** | **Database** | **Country** |
| --- | --- | --- | --- | --- |
| ***Amazona*** | *ochrocephala* | PQ345171 | NCBI | Colombia |
|  | *ochrocephala* | PQ345178 | NCBI | Colombia |
|  | *ochrocephala* | BSPBA298-07 | BOLD System | Panama |
|  | *aestiva* | FJ027054 | NCBI | Argentina |
|  | *aestiva* | FJ027053 | NCBI | Argentina |
|  | *barbadensis* | JX524615 | NCBI | Not reported |
|  | *amazonica* | PQ345091 | NCBI | Colombia |
|  | *guildingii* | JQ174003 | NCBI | Saint Vincent and the Grenadines |
|  | *autumnalis* | OR537571 | NCBI | Colombia |
|  | *autumnalis* | JQ173996 | NCBI | Panama |
|  | *autumnalis* | PQ345282 | NCBI | Colombia |
|  | *finschi* | BOM1412-10 | BOLD System | Mexico |
|  | *finschi* | BOM1409-10 | BOLD System | Mexico |
|  | *leucocephala* | OR048934 | NCBI | Cuba |
|  | *leucocephala* | OR048930 | NCBI | Cayman Islands |
|  | *ventralis* | NC034679 | NCBI | Not reported |
| ***Ara*** | *ararauna* | OR537569 | NCBI | Colombia |
|  | *ararauna* | OR537570 | NCBI | Colombia |
|  | *glaucogularis* | NC026029 | NCBI | Bolivia |
|  | *macao* | JQ174064 | NCBI | Brazil |
|  | *macao* | DTGIH087-24 | BOLD System | Colombia |
|  | *macao* | PCMED014-24 | BOLD System | Colombia |
|  | *militaris* | NC027839 | NCBI | Not reported |
|  | *ambiguus* | NC082166 | NCBI | Not reported |
|  | *chloropterus* | KR017982 | NCBI | Canada |
|  | *chloropterus* | KF525375 | NCBI | Canada |
|  | *tricolor* | NC037895 | NCBI | Cuba |
|  | *tricolor* | MG432917 | NCBI | Cuba |
|  | *severus* | OR537573 | NCBI | Colombia |
|  | *severus* | KF446114 | NCBI | Colombia |
|  | *severus* | JQ174066 | NCBI | Brazil |

**Supplementary Table S2**. External 16S rRNA reference sequences used in phylogenetic and barcoding analyses.

| **Genus** | **Species** | **Accession number** | **Database** | **Country** |
| --- | --- | --- | --- | --- |
| ***Amazona*** | *barbadensis* | JX524615.1 | NCBI | Not reported |
|  | *auropalliata* | NC_063712.1 | NCBI | Not reported |
|  | *ochrocephala* | NC_027840.1 | NCBI | Not reported |
|  | *aestiva* | NC_033336.1 | NCBI | Brazil |
|  | *autumnalis* | PQ068723.1 | NCBI | Colombia |
|  | *leucocephala* | OR048931 | NCBI | Bahamas |
|  | *leucocephala* | OR048943 | NCBI | Bahamas |
|  | *leucocephala* | OR048935 | NCBI | Turks and Caicos Island |
|  | *ventralis* | OR048929 | NCBI | Haiti |
|  | *ventralis* | OR0489237 | NCBI | Dominican Republic |
|  | *guildingii* | NC_052783.1 | NCBI | Not reported |
|  | *vinacea* | NC_080983 | NCBI | Not reported |
| ***Ara*** | *ararauna* | OR819871 | NCBI | Colombia |
|  | *ararauna* | OR819873 | NCBI | Colombia |
|  | *rubrogenys* | PQ436350 | NCBI | Not reported |
|  | *severus* | OR819874 | NCBI | Colombia |
|  | *severus* | PQ068719 | NCBI | Colombia |
|  | *militaris* | JX524613 | NCBI | Not reported |
|  | *militaris* | PQ068725 | NCBI | Colombia |
|  | *militaris* | PQ436348 | NCBI | Not reported |
|  | *ambiguus* | PQ167824 | NCBI | Not reported |
|  | *ambiguus* | PQ068722 | NCBI | Colombia |
|  | *macao* | MW584234 | NCBI | Peru |
|  | *macao* | MW584236 | NCBI | Peru |
|  | *macao* | PQ167823 | NCBI | Not reported |

**Supplementary Table S3**. Genetic differentiation and diversity indices estimated from COI and 16S rRNA sequences in individuals of the genera *Amazona* and *Ara*.

|  | *Amazona* spp. | | *Ara* spp. | |
| --- | --- | --- | --- | --- |
|  | **COI gene** | **16S rRNA gene** | **COI gene** | **16S rRNA gene** |
| Genetic diversity indices | | | | |
| Fst | 0.9608 | 0.0017 | 0.9630 | 0.7455 |
| Hs | 0.0128 | 0.1663 | 0.0113 | 0.0767 |
| Ht | 0.3254 | 0.1666 | 0.3047 | 0.3013 |

**Supplementary Table S4**. Specimen information and GenBank and BOLD accession numbers for COI sequences generated in this study.

| Animal ID | Seizure region | GenBank accession number | BOLD accession number |
| --- | --- | --- | --- |
| AMVA01 | Antioquia | PV130528 | UNCUR018-25 |
| AMVA02 | Antioquia | PV130565 | UNCUR055-25 |
| AMVA03 | Antioquia | PV130566 | UNCUR056-25 |
| AMVA05 | Antioquia | PV130553 | UNCUR043-25 |
| AMVA06 | Antioquia | PV130564 | UNCUR054-25 |
| AMVA08 | Antioquia | PV130517 | UNCUR007-25 |
| AMVA09 | Antioquia | PV130558 | UNCUR048-25 |
| AMVA10 | Antioquia | PV130552 | UNCUR042-25 |
| AMVA12 | Antioquia | PV130515 | UNCUR005-25 |
| AMVA13 | Antioquia | PV130557 | UNCUR047-25 |
| AMVA14 | Antioquia | PV130551 | UNCUR041-25 |
| AMVA15 | Antioquia | PV130550 | UNCUR040-25 |
| AMVA16 | Antioquia | PV130570 | UNCUR060-25 |
| AMVA17 | Antioquia | PV130563 | UNCUR053-25 |
| AMVA18 | Antioquia | PV130561 | UNCUR051-25 |
| AMVA20 | Antioquia | PV130560 | UNCUR050-25 |
| AMVA21 | Antioquia | PV130549 | UNCUR039-25 |
| AMVA23 | Antioquia | PV130548 | UNCUR038-25 |
| AMVA24 | Antioquia | PV130562 | UNCUR052-25 |
| AMVA25 | Antioquia | PV130547 | UNCUR037-25 |
| AMVA26 | Antioquia | PV130516 | UNCUR006-25 |
| AMVA27 | Antioquia | PV130546 | UNCUR036-25 |
| AMVA28 | Antioquia | PV130556 | UNCUR046-25 |
| AMVA29 | Antioquia | PV130545 | UNCUR035-25 |
| AMVA30 | Antioquia | PV130569 | UNCUR059-25 |
| AMVA31 | Antioquia | PV130530 | UNCUR020-25 |
| AMVA32 | Antioquia | PV130514 | UNCUR004-25 |
| AMVA33 | Antioquia | PV130559 | UNCUR049-25 |
| AMVA36 | Antioquia | PV130544 | UNCUR034-25 |
| AMVA37 | Antioquia | PV130532 | UNCUR022-25 |
| AMVA38 | Antioquia | PV130529 | UNCUR019-25 |
| CC02 | Cesar | PV130542 | UNCUR032-25 |
| CC03 | Cesar | PV130541 | UNCUR031-25 |
| CC07 | Cesar | PV130526 | UNCUR016-25 |
| CC09 | Cesar | PV130527 | UNCUR017-25 |
| CC10 | Cesar | PV130540 | UNCUR030-25 |
| CC11 | Cesar | PV130543 | UNCUR033-25 |
| CC12 | Cesar | PV130525 | UNCUR015-25 |
| CC13 | Cesar | PV130524 | UNCUR014-25 |
| CC14 | Cesar | PV130539 | UNCUR029-25 |
| CC15 | Cesar | PV130538 | UNCUR028-25 |
| CC16 | Cesar | PV130523 | UNCUR013-25 |
| CC17 | Cesar | PV130522 | UNCUR012-25 |
| CC18 | Cesar | PV130521 | UNCUR011-25 |
| CC19 | Cesar | PV130520 | UNCUR010-25 |
| CC20 | Cesar | PV130519 | UNCUR009-25 |
| CVC01 | Valle del Cauca | PV130531 | UNCUR021-25 |
| CVC02 | Valle del Cauca | PV130535 | UNCUR025-25 |
| CVC04 | Valle del Cauca | PV130534 | UNCUR024-25 |
| CVC06 | Valle del Cauca | PV130512 | UNCUR002-25 |
| CVC08 | Valle del Cauca | PV130518 | UNCUR008-25 |
| CVC10 | Valle del Cauca | PV130568 | UNCUR058-25 |
| CVC11 | Valle del Cauca | PV130536 | UNCUR026-25 |
| CVC17 | Valle del Cauca | PV130567 | UNCUR057-25 |
| CVC18 | Valle del Cauca | PV130555 | UNCUR045-25 |
| CVC19 | Valle del Cauca | PV130554 | UNCUR044-25 |
| CVC22 | Valle del Cauca | PV130513 | UNCUR003-25 |
| CVC23 | Valle del Cauca | PV130511 | UNCUR001-25 |
| CVC25 | Valle del Cauca | PV130537 | UNCUR027-25 |
| CVC26 | Valle del Cauca | PV130533 | UNCUR023-25 |
|  |  |  |  |
|  |  |  |  |
|  |  |  |  |

**Supplementary Table S5**. Specimen information and GenBank accession numbers for 16S rRNA sequences generated in this study.

| **Animal ID** | **Seizure region** | **GenBank accession number** |
| --- | --- | --- |
| AMVA01 | Antioquia | PV131656 |
| AMVA03 | Antioquia | PV131635 |
| AMVA04 | Antioquia | PV131610 |
| AMVA05 | Antioquia | PV131600 |
| AMVA06 | Antioquia | PV131632 |
| AMVA07 | Antioquia | PV131659 |
| AMVA08 | Antioquia | PV131649 |
| AMVA09 | Antioquia | PV131631 |
| AMVA10 | Antioquia | PV131608 |
| AMVA11 | Antioquia | PV131658 |
| AMVA12 | Antioquia | PV131650 |
| AMVA13 | Antioquia | PV131634 |
| AMVA14 | Antioquia | PV131601 |
| AMVA16 | Antioquia | PV131646 |
| AMVA17 | Antioquia | PV131636 |
| AMVA18 | Antioquia | PV131637 |
| AMVA20 | Antioquia | PV131639 |
| AMVA21 | Antioquia | PV131609 |
| AMVA23 | Antioquia | PV131607 |
| AMVA24 | Antioquia | PV131638 |
| AMVA25 | Antioquia | PV131604 |
| AMVA26 | Antioquia | PV131651 |
| AMVA27 | Antioquia | PV131602 |
| AMVA29 | Antioquia | PV131603 |
| AMVA30 | Antioquia | PV131647 |
| AMVA31 | Antioquia | PV131674 |
| AMVA32 | Antioquia | PV131652 |
| AMVA34 | Antioquia | PV131633 |
| AMVA35 | Antioquia | PV131605 |
| AMVA36 | Antioquia | PV131606 |
| AMVA37 | Antioquia | PV131675 |
| AMVA38 | Antioquia | PV131657 |
| CC01 | Cesar | PV131618 |
| CC02 | Cesar | PV131612 |
| CC03 | Cesar | PV131611 |
| CC05 | Cesar | PV131615 |
| CC07 | Cesar | PV131662 |
| CC09 | Cesar | PV131663 |
| CC10 | Cesar | PV131614 |
| CC11 | Cesar | PV131613 |
| CC12 | Cesar | PV131664 |
| CC13 | Cesar | PV131665 |
| CC14 | Cesar | PV131617 |
| CC15 | Cesar | PV131616 |
| CC16 | Cesar | PV131666 |
| CC17 | Cesar | PV131667 |
| CC18 | Cesar | PV131668 |
| CC19 | Cesar | PV131669 |
| CC20 | Cesar | PV131670 |
| CVC01 | Valle del Cauca | PV131671 |
| CVC02 | Valle del Cauca | PV131619 |
| CVC03 | Valle del Cauca | PV131622 |
| CVC04 | Valle del Cauca | PV131620 |
| CVC05 | Valle del Cauca | PV131648 |
| CVC06 | Valle del Cauca | PV131653 |
| CVC07 | Valle del Cauca | PV131621 |
| CVC08 | Valle del Cauca | PV131661 |
| CVC09 | Valle del Cauca | PV131660 |
| CVC10 | Valle del Cauca | PV131643 |
| CVC11 | Valle del Cauca | PV131623 |
| CVC12 | Valle del Cauca | PV131642 |
| CVC13 | Valle del Cauca | PV131626 |
| CVC15 | Valle del Cauca | PV131624 |
| CVC16 | Valle del Cauca | PV131645 |
| CVC17 | Valle del Cauca | PV131644 |
| CVC18 | Valle del Cauca | PV131640 |
| CVC19 | Valle del Cauca | PV131641 |
| CVC20 | Valle del Cauca | PV131625 |
| CVC22 | Valle del Cauca | PV131655 |
| CVC23 | Valle del Cauca | PV131654 |
| CVC24 | Valle del Cauca | PV131673 |
| CVC25 | Valle del Cauca | PV131629 |
| CVC26 | Valle del Cauca | PV131627 |
| CVC27 | Valle del Cauca | PV131630 |
| CVC28 | Valle del Cauca | PV131672 |
| CVC29 | Valle del Cauca | PV131628 |
